# Peptide-Functionalized Liposomal Nanocarriers for Targeted Therapy of Liver Fibrosis and Hepatocellular Carcinoma: Design, Mechanisms, and Clinical Prospects

**DOI:** 10.1021/acsptsci.5c00719

**Published:** 2026-01-23

**Authors:** Kashif Maroof, Ronald Fook Seng Lee, Pinar Karacabey, Rükan Genç

**Affiliations:** † SUNUM, Sabanci University Nanotechnology Research Centre, 52991 Sabanci University, Istanbul TR-34956, Turkey; ‡ School of Pharmacy, 65210 Monash University Malaysia, 47500 Bandar Sunway, Selangor Darul Ehsan, Malaysia; § Department of Chemical Engineering Faculty of Engineering, 52983 Mersin University, Mersin 33343, Turkey

**Keywords:** liposomes, peptide targeting, hepatocellular carcinoma, liver fibrosis, nanomedicine, drug delivery

## Abstract

Liver fibrosis and hepatocellular carcinoma (HCC) remain major global health burdens, in part due to limited drug specificity, off-target toxicity, and the complex hepatic microenvironment. Peptide-functionalized liposomal nanocarriers have emerged as a promising approach to enhance cell-selective drug delivery to activated hepatic stellate cells in fibrosis and malignant hepatocytes in HCC. This review critically examines recent progress in peptide-guided liposomal systems, focusing on design strategies, receptor-mediated targeting mechanisms, and translational considerations. Key peptide ligands, including cyclic RGD peptides targeting integrins αvβ3/αvβ5, GE11 for epidermal growth factor receptor, and transferrin receptor-binding peptides, are discussed in relation to their roles in promoting receptor-mediated endocytosis. Liposome fabrication methods and ligand conjugation chemistries are evaluated for their impact on stability, ligand presentation, and in vivo biodistribution. Preclinical evidence demonstrating improved drug accumulation, reduced fibrosis markers, and suppression of tumor growth is summarized alongside current limitations including receptor heterogeneity, extracellular matrix barriers, and manufacturing scalability. Finally, emerging directions such as stimuli-responsive and theranostic liposomes as well as combination strategies with immunomodulatory therapies are highlighted. By integrating mechanistic insight with design and translational perspectives, this review identifies key opportunities and the remaining hurdles in advancing peptide-targeted liposomal nanomedicines for liver disease.

## Graphical abstract

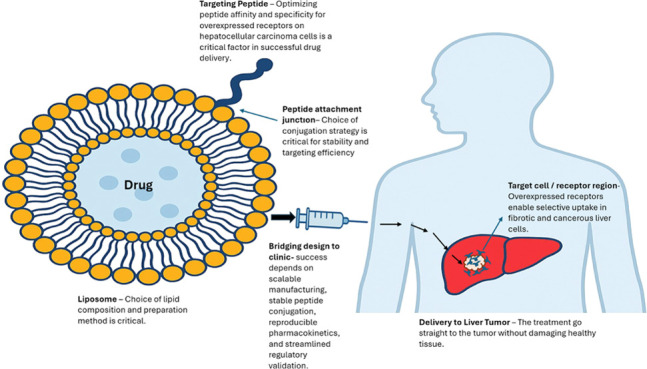

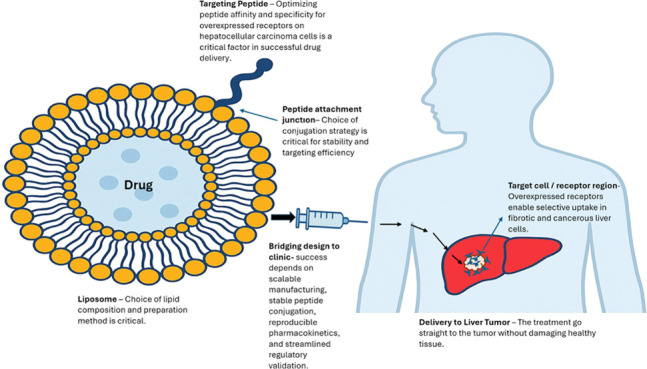

## 1 Introduction

The liver, the body’s largest internal organ, is essential for detoxification, protein synthesis, metabolic regulation, and immune defense.^1^ Its complex architecture and high vascularization make it uniquely vulnerable to a wide range of injuries, aggressions, or pathogenic factors, including viral infections, alcohol abuse, and metabolic disorders.^2^ Chronic liver injury often triggers an aberrant wound healing response known as liver fibrosis, characterized by excessive extracellular matrix deposition and activation of hepatic stellate cells (HSCs).^3^ If left untreated, fibrosis can progress to cirrhosis and hepatocellular carcinoma (HCC), the most common and lethal form of primary liver cancer.^4–6^ Figure 1 illustrates the stages of chronic liver disease.

**Figure 1:**
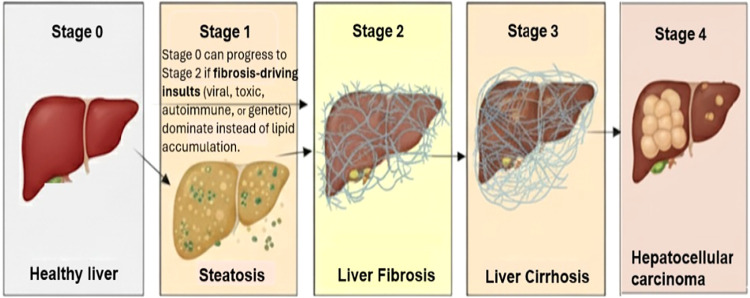
Progression of Chronic Liver Disease from Healthy Liver to HCC The figure illustrates the five stages of chronic liver disease. Stage 0 represents a healthy liver with a normal architecture. Disease begins with Stage 1, Steatosis (fatty liver), where the liver is yellowish and enlarged. While isolated steatosis is usually benign and reversible, the presence of inflammation (e.g., NASH or ASH) is concerning. Stage 2 marks the start of fibrosis, where a thin scar tissue (collagen) network forms, giving the liver an irregular surface; early fibrosis can be partly reversible. Stage 3 involves Advanced Fibrosis with thick scar bands and nodular regeneration, resulting in a hard, functionally impaired liver. This stage is generally irreversible. The disease culminates in Stage 4, Hepatocellular Carcinoma (HCC), characterized by one or multiple tumor nodules often developing within the cirrhotic tissue.

The rationale for targeted drug delivery in liver disease is strongly supported by disease-specific differences in cellular composition and surface receptor expression across hepatic pathologies.^7^ In metabolic liver disorders such as hepatic steatosis and nonalcoholic steatohepatitis (NASH), hepatocytes represent the primary therapeutic target, with Kupffer cells (KCs) also contributing to disease progression through inflammatory signaling.^8^ Hepatocytes abundantly express the asialoglycoprotein receptor (ASGPR), a well-established endocytic receptor that recognizes galactose- and N-acetylgalactosamine (GalNAc)-containing ligands.^7^ Exploiting ASGPR-mediated uptake enables highly selective hepatocyte targeting and has therefore been widely explored for the delivery of nucleic acids and small-molecule therapeutics in steatosis and NASH.^7^

In chronic liver injury leading to fibrosis and cirrhosis, activated aHSCs are the principal effector cells responsible for excessive extracellular matrix deposition.^9^ Upon activation, HSCs upregulate several surface receptors, including integrins (notably αvβ3 and αvβ5), platelet-derived growth factor receptor-β (PDGFR-β), and the mannose-6-phosphate receptor (M6PR).^10–12^ These receptors have been extensively leveraged for active targeting using ligands such as linear or cyclic peptides, PDGFR-binding peptides, and M6P-derived motifs, enabling preferential accumulation of therapeutic payloads within fibrotic regions while sparing healthy parenchyma.^13–15^

Hepatocellular carcinoma further expands the repertoire of exploitable molecular targets due to malignant transformation and tumor-associated angiogenesis. In HCC, both cancerous hepatocytes and the tumor vasculature overexpress receptors including integrins (particularly αvβ3), epidermal growth factor receptor (EGFR/HER1), transferrin receptor (TfR), and glypican-3 (GPC3).^15–18^ Peptides such as RGD, GE11 (an EGFR-binding ligand), transferrin-mimetic peptides, and GPC3-targeting sequences have demonstrated enhanced tumor accumulation and receptor-mediated internalization in preclinical models.^19–21^ Targeting tumor cells through integrins or vascular EGFRs additionally offers an indirect but effective strategy to disrupt angiogenesis and improve drug penetration within HCC lesions.^22^

Beyond fibrotic and malignant conditions, inflammatory liver diseases present distinct opportunities for targeting immune–hepatocyte interactions.^23^ Chemokine receptors such as CXCR4 and other G protein–coupled receptors (GPCRs) are upregulated on immune cells and hepatocytes during chronic inflammation, making them attractive targets for peptide-based antagonists aimed at modulating immune cell recruitment and inflammatory signaling pathways.^24^

Finally, in rare but clinically relevant neuroendocrine liver tumors, somatostatin receptors particularly SSTR2 and SSTR5 are highly overexpressed and exhibit rapid ligand-induced internalization.^25^ This biological property has been successfully exploited by somatostatin analog peptides for both diagnostic imaging and targeted therapy, underscoring the broader applicability of receptor-guided delivery strategies within hepatic malignancies.^26^

Collectively, these disease- and cell-specific receptor profiles provide a strong biological foundation for the design of peptide-functionalized liposomal nanocarriers, enabling selective targeting across the spectrum of liver diseases while addressing the limitations of conventional systemic therapies.

Nanocarrier-based drug delivery platforms, particularly liposomes (10–200 nm), have emerged as promising solutions to these challenges.^27^ Composed of phospholipid bilayers, liposomes offer high biocompatibility and can encapsulate both hydrophilic and hydrophobic drugs. Their tunable surface properties allow for targeted delivery, while their structure enhances drug solubility, stability, and circulation half-life. These features collectively improve pharmacokinetics and reduce systemic toxicity, making liposomes highly effective for addressing the complex demands of liver disease treatment.^27^ FDA-approved liposomal formulations (e.g., Doxil) exemplify their clinical impact, demonstrating reduced cardiotoxicity and enhanced tumor accumulation of chemotherapeutics like doxorubicin.^28^ In liver cancer, liposomes exploit the enhanced permeability and retention (EPR) effect to passively target tumors,^29^ while in fibrosis, they can be actively directed to activated hepatic stellate cells (aHSCs) for localized antifibrotic delivery.^30^

To enhance the selectivity and performance of liposomal carriers, several targeting strategies have been explored, including passive targeting via PEGylation and active targeting through ligands such as peptides, antibodies, or small molecules.^31^ Among these, peptide-based targeting offers distinct advantages, including high specificity, low immunogenicity, and the ability to trigger receptor-mediated endocytosis.^32^ In fibrotic liver disease, peptides can be designed to target receptors overexpressed on activated HSCs such as integrins, PDGFR-β, and AT1R enabling selective drug delivery to the fibrotic niche.^33^ In HCC, targeting receptors like EGFR or αvβ3 integrins enhances tumor accumulation and cytotoxicity while sparing healthy tissue.^10^ Beyond drug delivery, these peptide-guided liposomal systems also show promise in diagnostics and theranostics. By incorporating imaging agents or dual-functional payloads, they enable early detection, disease monitoring, and personalized therapy in liver fibrosis and cancer.^34^ However, despite growing interest and numerous preclinical efforts, a consolidated understanding of how peptide-liposome systems can be rationally designed and translated specifically for liver-targeted applications remains limited.

This review is structured to examine peptide-functionalized liposomal systems for targeted hepatic therapy. Section 2 focuses on the design and formulation of liposomes for targeted therapeutics, including liver-specific targeting peptides, liposome preparation methods, and peptide conjugation strategies. Section 3 discusses liposomal therapy in the context of passive accumulation versus active targeting, with particular emphasis on peptide-targeted liposomes and receptor-mediated drug delivery through integrins, TfR receptors, HER-family receptors, and G protein–coupled receptors. Section 4 addresses the clinical development landscape of peptide-modified liposomes, highlighting translational barriers and clinical prospects for liver disease therapy. Finally, Section 5 outlines future directions and emerging trends in liposomal drug delivery systems for liver diseases.

## 2 Design and Formulation of Liposomes for Targeted Therapeutics

Liposomes are spherical vesicles composed primarily of phospholipids such as soybean phosphatidylcholine or synthetic dialkyl lipids that self-assemble into bilayered structures. Cholesterol is frequently incorporated to enhance membrane rigidity, reduce permeability, and stabilize the bilayer in physiological environments like blood and plasma.^35–37^ In liver-targeted formulations, cholesterol helps prevent premature drug leakage and supports structural integrity, particularly important for navigating fibrotic or tumor-altered liver tissues.^38^

To prolong circulation time and minimize rapid clearance by the reticuloendothelial system (RES), liposomes are often “stealth”-modified with poly­(ethylene glycol) (PEG), a strategy known as PEGylation. PEGylated liposomes exhibit improved pharmacokinetic profiles and can better penetrate complex hepatic microenvironments such as fibrotic septa HCC nodules.^27,39^

As illustrated in Figure 2a, liposomes are categorized by bilayer architecture into unilamellar vesicles (ULVs), multilamellar vesicles (MLVs), and multivesicular vesicles (MVVs). ULVs comprising a single lipid bilayer are further subclassified into small (SUVs), large (LUVs), and giant (GUVs) vesicles.^40^ MLVs, with multiple concentric bilayers, often exhibit higher drug-loading potential, while SUVs (typically <200 nm) offer superior tissue penetration and are generally preferred in liver-targeted applications due to their favorable biodistribution and uptake properties.^35^ Figure 2b further visualizes how the addition of cholesterol and surface modifications, like targeting peptides refine these structures for therapeutic use.

**Figure 2:**
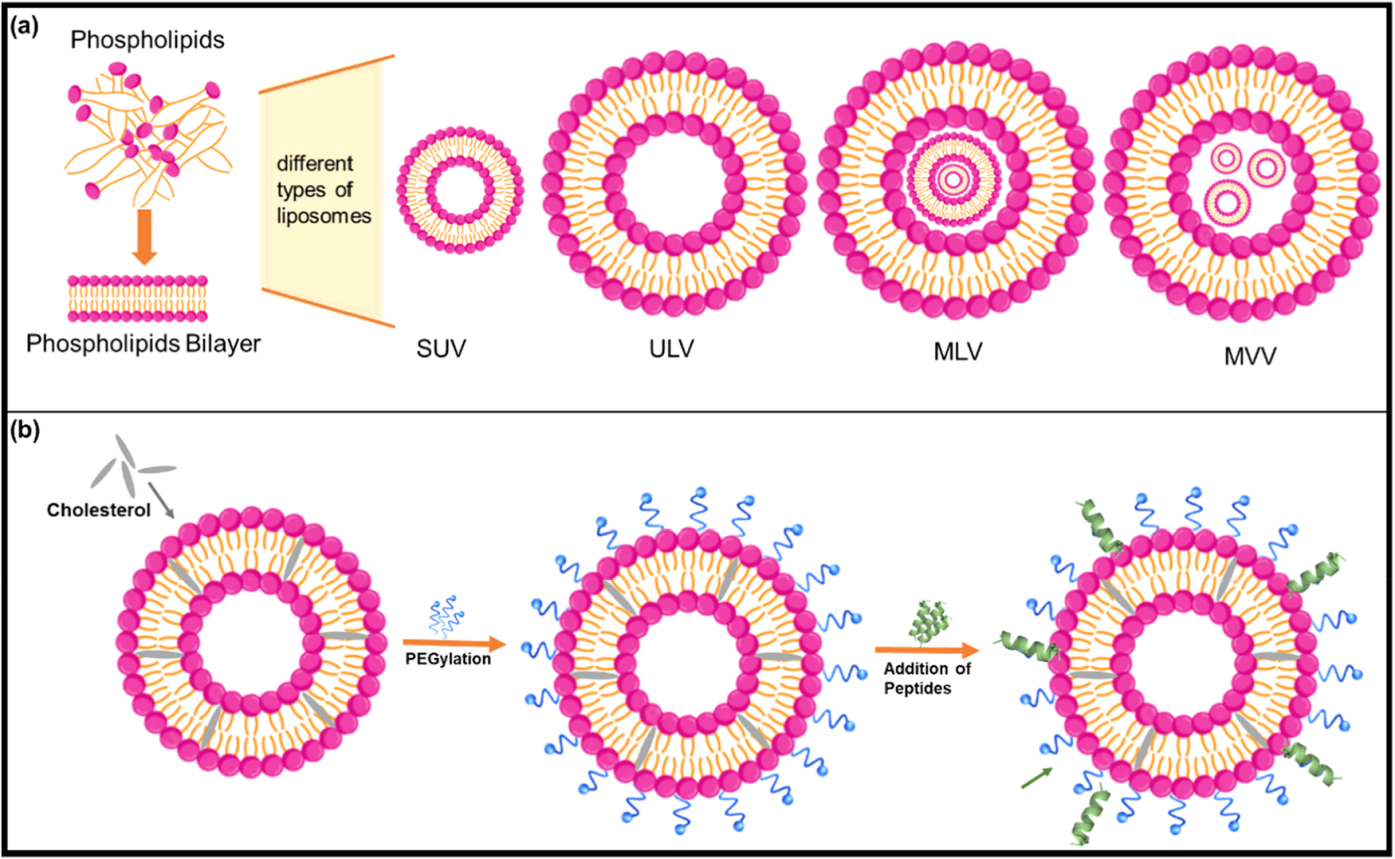
Structural organization and surface modification of liposomes (a) phospholipid bilayer assembly and vesicle types and (b) sequential modification with cholesterol, PEG, and peptide ligands.

A successful liver-targeted liposomal drug delivery system requires careful orchestration of three interconnected elements: the method of liposome preparation, the selection of targeting peptides, and the strategy used to conjugate those peptides onto the liposome surface.^27^ Preparation methods not only determine the size, lamellarity, and stability of liposomes but also dictate the feasibility and timing of ligand integration whether during lipid film formation or via postinsertion.^37^ The targeting peptides themselves must be stable, selective, and compatible with the disease context (e.g., HCC or fibrosis), while the conjugation chemistry must preserve peptide bioactivity and ensure durable attachment.^33^

The following sections examine each of these critical dimensions in detail. Section 2.1 outlines the major classes of targeting peptides explored in hepatic nanomedicine. Section 2.2 discusses commonly used liposome preparation techniques and their implications for liver-specific delivery. Section 2.3 presents an in-depth overview of peptide conjugation strategies, highlighting how different chemistries influence the orientation, stability, and targeting efficacy.

### 2.1 Targeting Peptides for Liver-Specific Drug Delivery

The success of peptide-functionalized liposomal systems in liver-targeted therapy hinges on the careful selection of peptide ligands with optimal stability, specificity, and functional performance.^41^ A diverse array of peptide types has been developed and explored for nanocarrier targeting, each offering distinct structural, biochemical, and pharmacological properties.^42^ These include linear and cyclic peptides, retro-inverso analogs, cell-penetrating peptides (CPPs), enzyme-activatable peptides, and those identified through high-throughput techniques like phage display. Understanding the unique characteristics and translational potential of each peptide class is critical to optimizing targeting precision and overcoming physiological barriers in hepatic diseases, such as fibrosis and HCC.

#### 2.1.1 Linear Peptides

Linear peptides represent the most straightforward class of targeting molecules, characterized by their unbranched, open-chain amino acid sequences.^41^ Their ease of synthesis and straightforward modification through standard solid-phase peptide synthesis make them highly appealing for initial screening and design.^41^ In the context of liver targeting, these peptides are typically engineered to selectively bind to receptors predominantly expressed on hepatocyte surfaces or other liver cell types, such as the asialoglycoprotein receptor (ASGR1), which is abundant on hepatocytes and involved in the clearance of desialylated glycoproteins.^43^ For example, peptides like SP94 (SFSIIHTPILPLGGC) and GE11 (YHWYGYTPQNVI) have been explored for their ability to target HCC by binding overexpressed receptors on liver cancer cells, enabling precise drug delivery.^20,44^ While their specific binding properties can facilitate effective localization to the liver, a significant challenge lies in their inherent susceptibility to degradation by proteases present in the bloodstream.^45^ This enzymatic breakdown can dramatically reduce their circulatory half-life, compromising their ability to reach the liver efficiently and bind to intended targets. For instance, linear peptides without structural stabilization (e.g., cyclization or D-amino acid substitutions) often exhibit rapid clearance, limiting their therapeutic window.^45^

#### 2.1.2 Cyclic Peptides

Cyclic peptides, such as cyclic Arginine-Glycine-Aspartic acid (cRGD) and cyclic Arginine-Glycine-Aspartic acid-tyrosine-Lysine (cRGDyK), offer significant advantages over linear peptides due to their constrained ring-like structure, which imparts greater conformational rigidity and spatial stability.^46,47^ This structural confinement reduces the peptide’s entropy loss upon receptor binding, often translating to higher binding affinity and selectivity for target receptors.^48^ Importantly, cyclic peptides demonstrate marked resistance to enzymatic degradation, particularly by exopeptidases and endopeptidases present in blood and tissue, thereby extending their plasma half-life and maintaining bioactivity *in vivo*.^49^

In liver-targeted nanomedicine, cRGD peptides have been extensively utilized for targeting integrin receptors, especially αvβ3, which are overexpressed on activated aHSCs in fibrotic livers and on tumor-associated vasculature in HCC.^10^ This makes them highly suitable ligands for the selective delivery of antifibrotic or anticancer agents via liposomal carriers. For example, studies using cRGD-functionalized liposomes have demonstrated enhanced accumulation in fibrotic liver regions, reduced fibrosis markers, and improved therapeutic indices compared to nontargeted formulations.^46^

Furthermore, the stability of cyclic peptides supports their incorporation into liposomal systems using preinsertion or postinsertion conjugation techniques, including maleimide–thiol coupling, without significant loss of targeting efficacy.^47,50^ Their compact size and defined structure also allow for precise ligand density tuning, which is crucial to avoid receptor saturation or immune recognition.^51^ As such, cyclic peptides represent a robust class of ligands for highly selective, stable, and efficient delivery of therapeutics to fibrotic and malignant hepatic tissues.^51^

#### 2.1.3 Retro-Inverso Peptides

Retro-inverso peptides are synthetic analogs in which the amino acids of a native peptide are substituted with their corresponding d-enantiomers.^52^ This approach preserves the spatial orientation of the side chains crucial for receptor recognition while inverting the peptide backbone.^52^ The resulting structure mimics the parent peptide’s biological activity but exhibits dramatically enhanced resistance to enzymatic degradation and longer systemic circulation due to the unnatural configuration of D-amino acids, which are poor substrates for endogenous proteases.^52^

This unique design makes retro-inverso peptides particularly attractive in nanomedicine, where the biological environment (e.g., serum proteases, acidic tumor microenvironments) can rapidly degrade conventional L-peptides.^52^ By enhancing *in vivo* half-life, retro-inverso peptides improve the therapeutic window and enable repeat dosing without loss of function.^52^

A prominent example is the DT7 peptide (hrpyiah), a retro-inverso analog of the LT7 peptide (HAIYPRH), which mimics transferrin and targets the TfR commonly overexpressed on HCC cells due to their high iron demand.^53^ Yu et al. developed DT7–DOX peptide–drug conjugates using a redox-sensitive disulfide linker. These conjugates selectively killed TfR-overexpressing tumor cells, while minimizing off-target toxicity. Notably, DT7–DOX outperformed its L-peptide counterpart (LT7–DOX) in terms of serum stability, controlled release, and therapeutic effect.^53^ In the context of liver-targeted liposomal systems, retro-inverso peptides like DT7 provide a robust platform for long-circulating, TfR-targeted delivery, making them a promising ligand class for liver cancer nanotherapy.^53^

#### 2.1.4 Cell-Penetrating Peptides (CPPs)

Cell-penetrating peptides (CPPs) are short, often cationic sequences capable of facilitating the translocation of macromolecules across cellular membranes through energy-independent mechanisms such as direct translocation or endocytic uptake.^54^ Classic examples include the HIV-1-derived transactivator of transcription (TAT) peptide and synthetic sequences like CPP44, both of which enhance the intracellular delivery of drug-loaded nanocarriers.^55,56^

CPPs, such as TAT and CPP44, facilitate intracellular drug delivery by enabling translocation across the cell membrane, independent of receptor-mediated endocytosis.^55,56^ While not inherently specific to liver tissues, CPPs can be combined with liver-targeting ligands to improve tumor penetration and drug release in fibrotic or cancerous microenvironments. In dual-ligand systems, CPPs often function synergistically with receptor-binding peptides to overcome transport barriers and heterogeneous receptor expression.^55,56^

#### 2.1.5 Enzyme-Activatable Peptides

Enzyme-activatable peptides remain inactive in systemic circulation and become functional only upon exposure to disease-specific enzymes such as matrix metalloproteinases (MMPs) or cathepsins abundantly expressed in the fibrotic or tumor microenvironment.^57^ These peptides are typically masked with a cleavable linker that is recognized and processed by enzymes present in diseased tissues, unveiling the active targeting sequence only at the site of action.^57^ In liver cancer models, MMP-2- or MMP-9-responsive peptides have been used to enhance the specificity of liposome uptake while reducing off-target effects, particularly useful in minimizing systemic toxicity of potent drugs.^58^

#### 2.1.6 Phage Display–Identified Targeting Peptides

Phage display is a high-throughput *in vitro* selection technique widely used to identify peptides with high affinity and specificity for disease-associated cellular targets, including receptors overexpressed in liver fibrosis and HCC.^59^ Unlike the preceding subsections, which classify peptides according to structural features (e.g., linear, cyclic, or retro-inverso), phage display represents a peptide discovery strategy rather than a structural category. As such, peptides identified through phage display can belong to multiple structural classes depending on their sequence composition and postselection modification.

Through screening of large combinatorial peptide libraries often comprising billions of unique sequences against liver cells, diseased tissues, or even whole animals (*in vivo* biopanning), phage display enables the isolation of ligands with precise tissue tropism and favorable binding characteristics.^59^ Notable examples include SP94, a linear peptide that selectively binds hepatocellular carcinoma cells, and DT7, a retro-inverso peptide designed to mimic transferrin binding to the transferrin receptor.^44,60^ Although these peptides are structurally classified as linear or retro-inverso, their identification through phage display has been central to their development as liver-targeting ligands.

When conjugated to liposomal nanocarriers, phage display–identified peptides such as SP94 and DT7 have demonstrated enhanced tumor accumulation and receptor-mediated uptake in preclinical liver cancer models.^44^ These examples illustrate how discovery-driven approaches can complement rational peptide design, expanding the repertoire of targeting ligands available for liver-specific liposomal drug delivery.

In summary, the landscape of targeting peptides for liver-specific liposomal delivery is rich and diverse, encompassing linear, cyclic, retro-inverso, cell-penetrating, enzyme-activatable, and phage display-derived ligands. Each class offers distinct structural and functional advantages, ranging from enhanced receptor selectivity and proteolytic stability to tunable intracellular trafficking. However, the therapeutic efficacy of these peptides is dependent not only on their biological affinity and specificity but also on how effectively they are conjugated to liposomal carriers. Table 1 provides a comparative overview of key classes of targeting peptides used in liver-specific liposomal drug delivery, highlighting their structural features, targeting mechanisms, stability profiles, and representative examples.

**Table 1:** Summary of Targeting Peptide Classes for Liver-Specific Liposomal Drug Delivery

peptide type	structural features	advantages	limitations	representative applications/examples	reference
linear peptides	open-chain, unbranched sequences	easy synthesis; customizable; good initial targeting	poor protease stability; rapid clearance	SP94 (targets HCC), GE11 (binds EGFR)	^20,44,61^
cyclic peptides	ring structure; constrained conformation	high receptor affinity; enhanced stability; reduced immune recognition	slightly more complex synthesis	cRGD, cRGDyK (target integrins on aHSCs and tumor vasculature)	^46,47^
retro-inverso peptides	D-amino acids in reverse sequence; preserves bioactive side chain orientation	protease resistance; extended circulation; mimics natural peptide activity	requires rational design and validation	DT7 (TfR-targeting in HCC), improved over LT7	^53,55^
cell-penetrating peptides (CPPs)	short, often cationic sequences (e.g., rich in Arg/Lys)	enhance cellular uptake; bypass receptor limitations; synergize with receptor ligands	nonspecific; may cause off-target effects	TAT, CPP44 (used in dual-ligand liposomes for tumor penetration)	^55^
enzyme-activatable peptides	peptides with cleavable linkers sensitive to disease-specific enzymes (e.g., MMPs)	disease-site activation; improved specificity; reduced systemic toxicity	require careful linker design; less studied in liver applications	MMP-2/MMP-9-cleavable peptides for tumor microenvironment–triggered liposome uptake	^58^
phage display-derived peptides	identified via high-throughput screening; often optimized for tissue/cell-specific binding	high specificity; customizable libraries; useful for difficult targets	may require postdiscovery stabilization/modification	SP94 (linear HCC-targeting peptide), DT7 (retro-inverso, TfR-binding peptide from *in vivo* biopanning)	^44,53,55^

### 2.2 Liposome Preparation Methods

In liver-targeted nanomedicine for HCC and fibrosis, selecting an appropriate liposome preparation technique is critical for therapeutic success.^62^ Several established and emerging methods are commonly used in experimental and preclinical studies, with each offering distinct advantages and limitations. This choice is particularly important when the preparation method must support subsequent conjugation of liver-specific targeting ligands, such as integrin- or transferrin-binding peptides, without compromising liposome integrity or performance.^63^

The initial synthesis of the plain liposome, which serves as the foundational nanocarrier, is typically achieved using methods such as thin-film hydration (the conventional Bangham method), ethanol injection, reverse-phase evaporation, or, more recently, highly controlled microfluidics-based techniques (Figure 3). After this initial step, the liposomal surface is functionalized to enable active targeting. Importantly, the preparation technique influences when and how peptide ligands are introduced either during liposome formation or through postassembly modification and directly affects particle size, surface characteristics, and drug retention.

**Figure 3:**
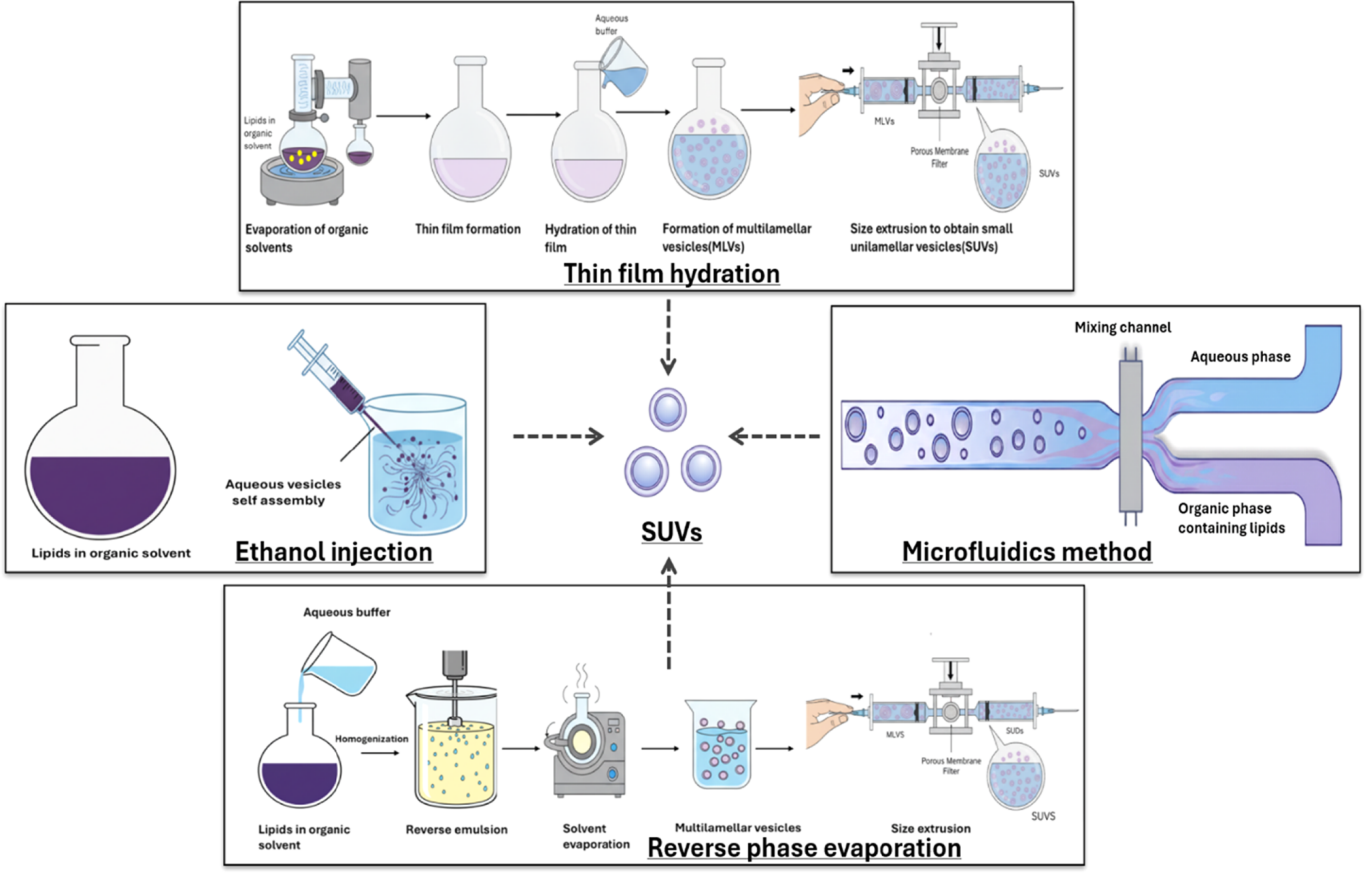
Foundational Liposome Preparation Techniques. Schematic representation of four primary methods for manufacturing liposomes (SUVs).

Three primary strategies are commonly employed for preparing peptide-targeted liposomes: the conventional method, the postinsertion method, and the postconjugation method.^64^ In the conventional method, liposomes are formed from a lipid mixture that already contains the targeting ligand preconjugated to a lipid anchor. This approach is typically implemented using thin-film hydration followed by extrusion, ensuring that the targeting moiety is incorporated into the lipid bilayer during liposome formation. As a result, this method offers a straightforward one-step process and often yields highly stable targeted nanocarriers.

In contrast, the postinsertion method follows a two-step process in which plain liposomes are first prepared and subsequently decorated with targeting ligands. The targeting peptide is commonly incorporated into micelles using a PEG–lipid conjugate and then incubated with preformed liposomes under elevated temperature conditions. This allows the lipid anchor to spontaneously insert into the existing bilayer. This strategy provides precise control over the ligand density on the liposomal surface and minimizes potential degradation of sensitive peptides during liposome fabrication.

The postconjugation method achieves targeting through covalent attachment of peptides to the liposome surface after liposome formation. This requires the inclusion of reactive functionalized lipids, such as those bearing carboxyl or amine groups, within the liposomal membrane. Following the activation of these functional groups, the targeting peptide is chemically coupled to the liposome surface. This approach results in a stable covalent bond, ensuring that the targeting ligand remains attached during circulation and offers the highest degree of surface stability.

Ultimately, the choice of preparation strategy must be guided by the stability requirements of both the encapsulated drug and the targeting peptide as well as the desired physicochemical and surface properties of the final therapeutic formulation. In the following section, we examine specific preparation methods applied in liver-specific peptide-functionalized liposomal systems, emphasizing how formulation decisions influence conjugation strategies and therapeutic performance.

#### 2.2.1 Thin-Film Hydration

The thin-film hydration technique remains one of the most utilized methods for peptide-modified liposome production, particularly in academic settings. This method involves dissolving lipids in an organic solvent (e.g., chloroform or methanol), evaporating the solvent to form a thin lipid film, and then hydrating it with an aqueous solution.^65^ MLVs are typically formed and further downsized into SUVs or LUVs by sonication or extrusion.^66^ In liver-targeted systems, this method has been widely employed due to its compatibility with PEGylation and postinsertion of peptide ligands.^67^

Du et al. and Li et al. both employed the thin-film hydration method to prepare cRGD peptide-modified liposomes for targeted drug delivery in liver fibrosis models, utilizing postinsertion of the targeting ligand.^47,50^ Du et al. formulated liposomes consisting of egg phosphatidylcholine (EPC), cholesterol (Chol), mPEG2000-DOPE, and maleimide-PEG2000-DOPE. Postinsertion of the targeting ligand was achieved by incubating the liposomes with cRGD peptide at a 10:1 molar ratio (lipid:peptide) overnight. This postinsertion approach enabled stable presentation of the cRGD peptide on the liposome surface without compromising encapsulation efficiency (EE) or particle stability. *In vitro* and *in vivo* studies demonstrated preferential uptake by aHSCs and achieved 10-fold greater accumulation in fibrotic liver tissue compared to nontargeted liposomes. In bile duct-ligated (BDL) rats, IFN-α1b-loaded cRGD liposomes significantly reduced fibrosis markers.^47^

Similarly, Li et al. formulated liposomes composed of EPC, cholesterol, mPEG2000-DOPE, and MAL-PEG2000-DOPE, hydrating the lipid film with Hepatocyte growth factor (HGF)-containing PBS before extrusion. Postinsertion of cRGD was performed under gentle stirring overnight again using a 10:1 lipid-to-peptide molar ratio. The resulting cRGD-functionalized liposomes showed significantly improved hepatic delivery of HGF, increased fibrosis reversal evidenced by reduced collagen and α-SMA expression, and prolonged systemic circulation of the encapsulated protein. Together, these studies highlight the consistency and efficacy of the thin-film hydration method combined with cRGD–maleimide–thiol chemistry to formulate integrin-targeted liposomes for liver fibrosis, offering a modular and clinically relevant strategy for antifibrotic drug delivery.^50^

Wu et al. also employed postinsertion strategy using SP94, a peptide specific for HCC cells, to functionalize PEGylated liposomes. Liposomes composed of distearoylphosphatidylcholine (DSPC), cholesterol, and mPEG2000-DSPE were prepared via thin-film hydration. Doxorubicin and vinorelbine were remotely loaded into the liposomes. SP94-PEG3400-DSPE was then postinserted into the preformed liposomes by incubation at 60 °C, the lipid’s phase transition temperature. *In vivo*, these SP94-modified liposomes demonstrated enhanced tumor-specific accumulation, improved drug retention, and superior antitumor efficacy in HCC-bearing mice compared to nontargeted formulations.^68^

Building on these examples, Cheng et al. utilized the thin-film hydration method to design pH-responsive liposomes for HCC therapy, incorporating both a hepatocyte-targeting peptide and a tumor-microenvironment-responsive linker.^58^ Their formulation included soy phosphatidylcholine (SPC), cholesterol, DSPE-PEG_1_k, DSPE-PEG_2_k, and MAL-PEG_2_k-DOPE, along with the chemotherapeutic agent 10-hydroxycamptothecin (HCPT). Unlike the previous studies that relied on postinsertion, peptide conjugation in this study was achieved during the lipid film formation step, allowing covalent attachment of a matrix metalloproteinase-2 (MMP-2)-cleavable peptide linker through maleimide–thiol chemistry prior to hydration. The results not only demonstrated the flexibility of the thin-film hydration method for cofunctionalization with both targeting and responsive elements but also highlighted the potential of integrating stimuli-sensitive components for achieving precision liver cancer therapy.

Taken together, these studies showcase the versatility and robustness of the thin-film hydration technique for formulating peptide-functionalized liposomes tailored to liver diseases. Whether through postinsertion of peptides (as in Du et al., Li et al., and Wu et al.) or preinsertion during lipid film formation (as in Cheng et al.), this method allows for modular design of liposomes with precise ligand presentation, controlled size via extrusion, and preservation of drug and peptide bioactivity. By enabling coincorporation of PEGylated lipids, responsive linkers, and targeting ligands, thin-film hydration continues to serve as a foundational platform in the development of advanced nanocarriers for fibrosis and HCC therapy.

#### 2.2.2 Ethanol and Solvent Injection

The ethanol injection method has emerged as a valuable technique for liver-targeted liposome preparation, offering advantages in simplicity, scalability, and reproducibility.^27^ In this approach, lipids dissolved in ethanol are rapidly injected into an aqueous phase, inducing spontaneous vesicle formation through solvent displacement.^69^ This process typically yields SUVs with low PDI and requires minimal postprocessing (e.g., no extrusion), making it ideal for reproducible formulations.^69^

While the ethanol injection method has seen widespread use in small-molecule drug delivery,^69^ fewer studies have specifically focused on peptide-functionalized liposomes for liver targeting using this approach. A recent study by Tang et al. demonstrated the development of GE11-modified paclitaxel and curcumin liposomes (CUR-PTX@GE11-L) using a two-step microfluidic-assisted preparation strategy for liver cancer therapy. Initially, CUR-PTX-loaded liposomes (CUR-PTX@L) were prepared by using microfluidic mixing of an ethanol-based lipid phase containing EPC, cholesterol, and DSPE-PEG2000 with an aqueous glucose solution. In the second step, postinsertion of the GE11 peptide was performed by mixing the preformed CUR-PTX@L with DSPE-PEG2000/DSPE-PEG2000-GE11 micelles using a microfluidic nanopreparator. This approach allowed for precise control over liposome size, surface composition, and ligand density. The final PEG/GE11-functionalized liposomes exhibited enhanced liver tumor targeting and improved antitumor efficacy *in vitro* and *in vivo*, highlighting the potential of microfluidic-assisted postinsertion methods for peptide-functionalized liposomal systems targeting HCC.^20^

Similarly, erianin-loaded liposomes (LP-ERN) were prepared using ethanol injection by dissolving 1,2-dioleoyl-3-trimethylammonium-propane, egg yolk phosphatidylcholine, cholesterol, and DSPE-mPEG2000 in ethanol at a molar ratio of 20:45:32:3. Erianin was codissolved in the lipid mixture at a concentration of 5 mg/mL and injected into phosphate-buffered saline (PBS) at a 1:10 ethanol-to-aqueous volume ratio under magnetic stirring, forming uniform liposomes without the need for extrusion. The resulting LP-ERN exhibited a particle size of 62.60 nm and a PDI of 0.137, with an EE of 69.5%, indicating excellent reproducibility and formulation stability. To enable tumor-specific delivery, transferrin functionalization was achieved via postinsertion. Transferrin was first thiolated using Traut’s reagent and conjugated to Mal-mPEG2000-DSPE, forming a Tf-PEG-lipid conjugate. This conjugate was incubated with LP-ERN at a 100:1 lipid-to-conjugate molar ratio at 37 °C for 30 min, allowing its integration into the liposomal membrane. The resulting Tf-modified liposomes (Tf-LP-ERN) showed a slightly increased particle size (88.63 nm) and PDI (0.165), while retaining a high EE of 68.5%. Notably, neither particle uniformity nor EE was compromised by the postinsertion process, and the modified formulation displayed superior tumor targeting and antiliver cancer activity in preclinical models.^70^

Together, these studies underscore the flexibility of the ethanol injection method as a platform for developing functionalized liposomal systems. When coupled with postinsertion techniques, it enables the precise integration of targeting ligands such as peptides and proteins without compromising the physicochemical integrity of the carrier. While challenges remain—such as preserving ligand bioactivity and ensuring deep tissue penetration, these advances demonstrate that ethanol injection is highly adaptable for liver-specific nanomedicine design, including applications in HCC and liver fibrosis.

#### 2.2.3 Reverse-Phase Evaporation

The reverse-phase evaporation (RPE) method is widely used for liposome production due to its ability to yield highly stable, drug-loaded vesicles with precise control over the lipid composition.^71^ This method involves dissolving lipids and other components in an organic solvent, which is then emulsified with an aqueous phase to form a reverse emulsion.^71^ Upon solvent evaporation, the emulsion collapses to form SUVs, which can encapsulate both hydrophobic and hydrophilic drugs effectively.^66^ RPE has proven particularly advantageous in the preparation of multifunctional liposomes, enabling the incorporation of magnetic nanoparticles or targeting ligands for enhanced drug delivery and site-specific targeting.^61,72^

Lin et al. developed paclitaxel-loaded magnetic polymeric liposomes conjugated with an EGFR-targeting peptide for HCC therapy. The liposomes were prepared by dissolving the OQC, PEG-OQC, cholesterol, and superparamagnetic iron oxide nanoparticles. Paclitaxel was coloaded during liposome formation. The resulting system demonstrated enhanced tumor accumulation up to 10-fold under external magnetic guidance and achieved a 75% reduction in tumor volume in HCC xenograft models.^72^

In contrast, Lin et al. took a more sophisticated approach by developing dual ligand modified liposomes using RPE for the targeted delivery of arsenic trioxide. The liposomes, composed of soy phosphatidylcholine (PC), cholesterol, and DSPE-PEG2000, were modified with both an anti-GPC3 antibody and a cell-penetrating peptide (CPP44) via postinsertion. The anti-GPC3 antibody targets glypican-3 on HCC cells, while CPP44 enhances intracellular drug delivery. This study underscores the versatility of RPE for creating liposomes with dual-targeting capabilities, allowing for enhanced specificity and cellular penetration for liver cancer treatment.^56^

These studies highlight the effectiveness of the RPE method in preparing peptide-functionalized liposomes with a high drug-loading capacity and precise targeting capabilities.

#### 2.2.4 Microfluidics-Based Preparation

Microfluidic technologies have emerged as a powerful alternative to conventional liposome fabrication methods, offering superior control over particle size, distribution, and surface functionality.^73^ In these systems, the self-assembly of liposomes is typically achieved by the rapid and controlled mixing of lipid-containing organic solvents with aqueous buffers within microchannels.^74^ Compared to bulk methods such as thin-film hydration or ethanol injection, microfluidics enables reproducible, scalable production of targeted nanocarriers under continuous flow, making it a compelling option for advanced liposomal drug delivery systems.^74^

While microfluidic techniques have been increasingly adopted for liposome preparation due to their scalability and precision, studies specifically applying this method for peptide-mediated liver-targeted liposomes remain limited. However, prior work in related systems demonstrates its feasibility and relevance. Ran et al. pioneered the use of microfluidic self-assembly to construct a combinatorial library of single- and dual-ligand liposomes for tumor-targeted applications. This platform enabled the precise incorporation of tumor-homing and CPPs during liposome formation, showcasing the potential for fine-tuning the ligand density and surface composition in real-time production. Although not liver-specific, the approach clearly supports complex, multifunctional liposome fabrication suitable for *in vivo* use.^75^

Building on this, Seleci et al. employed a rapid microfluidic mixing technique to prepare peptide-modified niosome vesicles based on nonionic surfactants. The study demonstrated successful peptide incorporation, narrow size distribution, and high vesicle stability.^76^ While niosomes differ structurally from phospholipid-based liposomes, the methodology and outcomes highlight transferable advantages for liposomal platforms, reinforcing microfluidics as a viable approach for developing peptide-functionalized nanocarriers with improved targeting precision.

As discussed in Section 2.2.2, Tang et al. further advanced this concept by developing GE11-modified paclitaxel and curcumin liposomes (CUR-PTX@GE11-L) using a two-step microfluidic-assisted strategy. The study exemplifies how microfluidics can be leveraged not only for initial liposome formation but also for fine-tuned ligand conjugation, resulting in stable PEGylated liposomes with enhanced liver tumor targeting and superior therapeutic efficacy. This highlights the versatility and translational promise of microfluidic approaches in the design of peptide-functionalized liposomes for HCC.

Taken together, these findings indicate that microfluidic fabrication holds significant promise for peptide-mediated liver-targeted liposome development despite the current lack of liver-specific studies. Further research is warranted to adapt microfluidic workflows specifically for liver-directed peptide ligands and to evaluate their therapeutic impact in relevant models.

#### 2.2.5 Extrusion and Size Control

Extrusion is frequently used as a postprocessing step to standardize liposome size. Liposomes are passed through polycarbonate membranes of defined pore sizes under pressure, producing ULVs with tight size distributions.^74^ This size control is crucial for solid tumor targeting in general, as liposomes below 200 nm (often <100 nm) exhibit improved accumulation via the EPR effect. In liver-targeted applications, especially HCC such dimensions also facilitate efficient passage through the fenestrated sinusoidal endothelium, enhancing tissue penetration and therapeutic efficacy.^77^

Nearly all the aforementioned studies employed extrusion to produce optimally sized liposomes for receptor targeting in fibrotic or cancerous liver tissue.^68,78^ Size control was essential for achieving cell-specific uptake, reducing RES clearance, and improving systemic pharmacokinetics.

The choice of liposome preparation method directly influences the success of peptide-functionalized systems for liver-targeted therapy. While traditional methods such as thin-film hydration and reverse-phase evaporation are widely used in experimental settings, microfluidics is emerging as a scalable platform suitable for clinical translation. Each method varies in terms of EE, reproducibility, and compatibility with surface modifications. For peptide-guided liver applications, particularly in HCC and liver fibrosis, selecting a method that ensures formulation stability, targeting precision, and efficient peptide conjugation is key to success.^79^

Table 2 provides a comparative overview of key liposome preparation methods used for developing peptide-functionalized liver-targeted drug delivery systems. It outlines each method’s distinctive features, major limitations, commonly employed peptide conjugation strategies, integration approaches (e.g., post- or preinsertion), and their respective target applications in liver diseases such as HCC and liver fibrosis. The table also highlights representative studies that exemplify the use of each method in this context. Collectively, this comparison helps clarify how the choice of preparation technique influences liposome size control, EE, surface modification compatibility, and ultimately, targeting performance in liver-specific nanomedicine. The preparation methods are assessed based on key functional attributes relevant to liver-targeted nanomedicine: ease of production (laboratory feasibility), conjugation versatility (range of peptide integration strategies supported), size control and stability (consistency of nanoscale vesicle production and colloidal stability), and scalability (potential for translation to clinical-scale manufacturing).

**Table 2:** Overview of Liposome Preparation Methods for Peptide-Functionalized Liver-Targeted Systems

preparation method	advantages	limitations	ease of production	size control and stability	scalability	peptide conjugation strategy	peptide integration	liver-targeting applications	key studies
thin-film hydration	widely used; flexible design; compatible with PEGylation and responsive linkers; supports controlled ligand orientation	requires extrusion/sonication for uniform size, lower drug loading for hydrophilic drugs	moderate	good (with extrusion)	moderate	maleimide–thiol	post- or preinsertion	HCC (SP94, GE11 peptides)	^10,47,50,68^
						NHS–ester amide bond formation		liver fibrosis (cRGD-modified liposomes)	
						EDC/NHS carbodiimide coupling		stimuli-responsive delivery	
						biotin–streptavidin linkage			
ethanol injection	scalable and reproducible; enables dual-drug coloading; compatible with micelle-based peptide insertion	limited peptide studies	high	excellent (<100 nm possible)	high	maleimide–thiol	postinsertion	HCC (GE11, transferrin-modified liposomes)	^18,20^
		risk of peptide instability in ethanol						codelivery of hydrophobic drugs.	
reverse-phase evaporation (RPE)	high encapsulation capacity; suited for multifunctional, dual-targeted systems	laborious	low	variable (requires optimization)	low	SPDP-mediated disulfide maleimide–thiol	postinsertion	HCC (dual-ligand systems: anti-GPC3 + CPP44)	^10,61^
		solvent removal must be optimized, scale-up complexity						magnetic-targeted delivery	
microfluidic self-assembly	precision in size and ligand ratio; suitable for high-throughput and clinical scale-up	specialized equipment	moderate–high	excellent (precise control)	moderate: scalable via parallelized microchip arrays rather than throughput	ligand-specific (varies)	inline or modular	emerging for HCC (GE11, combinatorial ligands)	^75,76^
		few liver-specific peptide studies						potential for dual-targeting	
extrusion (postprocessing)	achieves uniform vesicle size (<200 nm); critical for EPR effect and liver sinusoidal entry	does not enhance loading or conjugation directly, requires preformed liposomes	N/A	excellent size uniformity	N/A	N/A	postprocessing only	critical for HCC/fibrosis targeting (optimal size <200 nm)	^10,47,50,68^

Section 2.3 explores these conjugation strategies in detail, discussing how different chemical linkages influence peptide orientation, stability, and bioactivity on the liposome surface, ultimately shaping the performance of peptide-functionalized nanomedicines in liver disease.

### 2.3 Peptide Conjugation Strategies

The design of peptide-functionalized liposomes for liver disease therapy relies on robust, stable, and biocompatible conjugation techniques to ensure that the targeting peptide retains its activity while remaining firmly anchored to the liposomal surface.^62^ Several chemical strategies have been developed to facilitate peptide-lipid coupling, each with unique advantages depending on the properties of the peptide, liposome formulation, and therapeutic context.

#### 2.3.1 Maleimide–Thiol Coupling: A Gold-Standard Approach

Maleimide–thiol chemistry has emerged as a gold-standard strategy for site-specific conjugation of targeting peptides to liposomes, offering exceptional stability through covalent thioether bond formation.^80^ This approach involves incorporating maleimide-functionalized PEGylated lipids (e.g., DSPE-PEG2000-maleimide) into the liposomal bilayer.^81^ Depending on the formulation strategy and stability requirements, peptide conjugation can be performed either prior to liposome formation (preinsertion) or after liposome assembly (postinsertion), offering formulation flexibility while ensuring precision targeting. The maleimide groups subsequently react with thiol moieties typically introduced via terminal cysteine residues engineered into the peptide sequence, enabling controlled orientation and presentation of the targeting ligand.^82^ This precise conjugation method is particularly valuable for liver-directed therapies, where proper peptide orientation is critical for receptor engagement on target cells and receptor-mediated endocytosis.^83^

Du et al.^47^ and Li et al.^50^ leveraged maleimide–thiol chemistry to anchor cRGD peptides onto liposomes for liver fibrosis therapy. In each case, cRGD was conjugated to DSPE-PEG2000-maleimide and postinserted into preformed EPC/cholesterol liposomes, ensuring a stable thioether linkage that preserves integrin-binding activity during circulation. Du et al. observed liposomes preferentially targeted activated HSCs, with 10-fold higher accumulation in fibrotic liver tissue and significantly reduced liver fibrosis markers compared to nontargeted formulations, while Li et al. reported significantly enhanced hepatic accumulation of HGF, reduced collagen deposition, and improved liver function in cirrhotic rats. These parallel studies underscore the utility of maleimide–thiol chemistry coupled with cRGD targeting for effective, peptide-mediated delivery to fibrotic liver tissue.^47^

Building on these principles, Cheng et al. applied maleimide–thiol chemistry to engineer a hepatocyte-targeting liposomal formulation responsive to the tumor microenvironment for improved HCC therapy.^58^ The hepatocyte-specific peptide was conjugated via thiol–maleimide coupling and incorporated directly during the lipid film formation, rather than through postinsertion. *In vivo* studies demonstrated that these modified liposomes achieved enhanced accumulation in hepatic tumors and superior therapeutic efficacy compared to nontargeted systems, while minimizing systemic toxicity. Cheng’s work expands the application of maleimide–thiol chemistry beyond classical postinsertion, showing its adaptability in preinsertion schemes and in multifunctional designs that couple receptor-specific delivery with microenvironment responsiveness.

Tang et al. further demonstrated the versatility of maleimide–thiol chemistry in a microfluidic-assisted ethanol injection platform. In this study, DSPE-PEG2000-GE11, which was presynthesized via maleimide–thiol chemistry, was incorporated into liposomes coloaded with curcumin and paclitaxel. This streamlined one-step fabrication allowed for scalable preparation of peptide-functionalized liposomes, which showed enhanced delivery to EGFR-overexpressing HCC cells and improved *in vivo* antitumor efficacy. Importantly, this work expands the application of maleimide–thiol chemistry into microfluidic workflows and demonstrates its compatibility with dual drug delivery.^20^

Collectively, these studies reinforce the adaptability of maleimide–thiol coupling across diverse preparation methods, thin-film hydration, post- and preinsertion, and even microfluidic-assisted ethanol injection, highlighting its role as a clinically relevant strategy for precision-targeted liposomal drug delivery in liver disease.

#### 2.3.2 NHS–Ester Amide Bond Formation

NHS–ester chemistry provides a reliable and efficient approach for attaching targeting peptides to liposomal surfaces, offering stable amide bond formation between NHS-activated lipids and primary amine groups on peptides.^84^ This method is especially valuable in liver-targeted drug delivery, where preserving peptide bioactivity and achieving controlled ligand density are critical for effective receptor engagement.^84^ Ding et al. demonstrated this strategy by conjugating a CPP to DSPE-PEG2000-NHS under mild conditions, forming a stable amide linkage. Tissue distribution studies in rats revealed enhanced hepatic uptake of the CPP-modified liposomes compared to plain liposomes, with higher liver accumulation at 24 h and reduced off-target distribution to the spleen. These liver-specific improvements are particularly relevant for HCC therapy, where selective drug delivery is essential. The covalent peptide linkage ensured stability in circulation, while the functional surface modification enhanced cellular uptake.^78^

Another example involves the preparation of doxorubicin-loaded PEGylated liposomes composed of DSPC, cholesterol, and DSPE-PEG2000 by using thin-film hydration followed by extrusion. The SP94 peptide, which targets HCC cells, was conjugated via postinsertion of NHS-PEG-DSPE-SP94 into preformed liposomes at temperatures above the lipid phase transition. The resulting systems successfully achieved peptide densities of approximately 300–500 molecules per liposome, demonstrating efficient coupling and preserved liposomal integrity.^61^

Together, these studies demonstrate that NHS–ester-mediated peptide conjugation is a reliable and scalable technique for developing liver-targeted liposomes with enhanced cellular uptake and biodistribution, making it a promising approach for clinical translation in HCC therapy.

#### 2.3.3 Click Chemistry

Bio-orthogonal “click” chemistry reactions, particularly strain-promoted azide–alkyne cycloaddition (SPAAC) have emerged as powerful tools for site-specific peptide conjugation on liposomes.^85^ These reactions proceed with high efficiency and specificity under mild, aqueous, and catalyst-free conditions, preserving the structural integrity of both the liposomal membrane and sensitive biomolecules such as peptides.^85^ In SPAAC, azide-functionalized lipids react with cyclooctyne- or alkyne-functionalized peptides to form stable triazole linkages, enabling covalent and irreversible attachment.^85^

Compared to traditional conjugation strategies (e.g., maleimide–thiol chemistry), SPAAC eliminates the need for metal catalysts such as Cu­(I), which can be cytotoxic or interfere with biological function.^86^ Moreover, the modular nature of click chemistry allows for precise control over ligand density, spatial orientation, and valency on the liposomal surface, key parameters that govern receptor targeting efficiency and downstream cellular responses.^87^ This level of control makes SPAAC particularly attractive for designing next-generation targeted liposomal drug delivery systems with reproducible performance.

The study by Lu et al. remains highly relevant in the context of click chemistry-based liver targeting.^88^ The authors utilized SPAAC to modify mesenchymal stem cell-derived small extracellular vesicles (sEVs) with a liver-targeting single-chain variable fragment (scFv) against the asialoglycoprotein receptor (ASGR1). This was achieved through metabolic glycoengineering using Ac_4_ManNAz to introduce azide groups onto the sEV surface, followed by conjugation with DBCO-tagged scFv, which enabled highly specific hepatocyte targeting. The resulting CAR-sEVs showed significantly enhanced therapeutic efficacy in acetaminophen-induced acute liver failure, as evidenced by reduced liver enzyme levels, mitigated tissue damage, and stimulated hepatocyte proliferation.^88^ While this platform is nonliposomal, it clearly demonstrates the clinical potential of SPAAC click chemistry for targeted delivery in liver diseases. These findings underscore the underexplored potential of click chemistry for peptide-mediated liposomal drug delivery in liver-targeted therapies, particularly for enhancing the targeting precision, therapeutic index, and biocompatibility.

#### 2.3.4 EDC/NHS Carbodiimide Coupling

The EDC/NHS carbodiimide coupling method is commonly used to couple peptides containing carboxyl groups to liposomes with available amine-functionalized headgroups.^89^ This approach is particularly effective when peptides naturally possess terminal carboxyl groups, eliminating the need for additional modifications.^90^ A notable example is the study by Jiang et al., where the researchers utilized the EDC/NHS carbodiimide coupling method to attach glycyrrhetinic acid (GA) to liposomes.^91^ In this case, EDC was used to activate the carboxyl groups of GA, while NHS stabilized the activated ester, enabling efficient conjugation to the liposome surface. Their study showed that the GA-modified liposomes, when loaded with curcumin and combretastatin A4 phosphate (CA4P), exhibited enhanced cellular uptake, cytotoxicity, and antitumor activity compared to those of free drugs or unmodified liposomes. This demonstrates the effectiveness of the EDC/NHS coupling method in creating targeted delivery systems for liver cancer treatment.^91^

#### 2.3.5 Biotin–Streptavidin Linkage

The biotin–streptavidin linkage provides a highly stable yet reversible approach for conjugating targeting peptides to liposomes, leveraging the ultrahigh affinity between biotin and streptavidin.^92^ This system has proven to be valuable for modular screening of targeting peptides, as exemplified by the T7 phage p17 peptide, which demonstrated superior binding to heparan sulfate proteoglycans (HSPGs), a marker overexpressed in liver cancers. In preclinical models, biotinylated p17 peptide coupled to streptavidin-coated liposomes achieved more than 5-fold higher cellular uptake in HCC models compared to nontargeted formulations, underscoring its potential for liver-directed therapies.^93^ Despite its advantages, the system faces notable translational limitations, including potential streptavidin immunogenicity and risks of *in vivo* dissociation, which may compromise therapeutic stability and efficacy.^93^

#### 2.3.6 Disulfide-Based Conjugation Strategies

Disulfide bond formation offers a reversible, redox-sensitive strategy for peptide conjugation, particularly useful in tumor-targeted delivery where intracellular reducing environments can trigger ligand release or payload activation.^72^ In a study by Lin et al., paclitaxel-loaded magnetic polymeric liposomes (MPLs) were conjugated with an EGFR-targeting peptide using N-succinimidyl 3-(2-pyridyldithio) propionate (SPDP), a heterobifunctional cross-linker that introduces a pyridyl disulfide group onto the liposome surface. The MPLs were first treated with SPDP in phosphate buffer to install reactive disulfide handles, followed by incubation with the cysteine-containing EGFR peptide, forming a stable disulfide linkage between the peptide and the liposomal surface. This approach enabled efficient surface functionalization while preserving the liposome’s structural integrity and drug payload. *In vivo*, EGFR peptide-conjugated MPLs demonstrated enhanced tumor accumulation under external magnetic guidance and achieved a significant tumor volume reduction in HCC xenograft models. This study highlights the potential of disulfide-based conjugation for functionalizing complex liposomal systems, particularly those integrating targeting ligands with magnetic or responsive elements for multimodal liver cancer therapy.^72^

Table 3 summarizes the most commonly used peptide conjugation strategies in liver-targeted liposomal drug delivery, highlighting their underlying chemistry, operational requirements, key features, and representative applications. Comparative rankings are classified as High/Favorable (●●●), Moderate (●●), or Low/Challenging (●) to reflect relative performance across five key domains: specificity, bond stability, simplicity, biocompatibility, and scalability. Symbol-based scoring is employed to enable rapid comparison of conjugation strategies across these translational parameters. In this framework, High/Favorable (●●●) corresponds to methods with minimal technical barriers, reliable performance, and high reproducibility; Moderate (●●) describes approaches achievable using standard laboratory procedures but requiring optimization; and Low/Challenging (●) refers to technically complex strategies involving demanding synthesis steps or inherent stability limitations.Comparative rankings are classified as High/Favorable (●●●), Moderate (●●), or Low/Challenging (●) to reflect relative performance across five key domains: specificity, bond stability, simplicity, biocompatibility, and scalability. Symbol-based scoring is employed to enable rapid comparison of conjugation strategies across these translational parameters. In this framework, High/Favorable (●●●) corresponds to methods with minimal technical barriers, reliable performance, and high reproducibility; Moderate (●●) describes approaches achievable using standard laboratory procedures but requiring optimization; and Low/Challenging (●) refers to technically complex strategies involving demanding synthesis steps or inherent stability limitations.

**Table 3:** Peptide Conjugation Strategies for Liver-Targeted Liposomal Drug Delivery^a^

				comparative scoring metrics						
conjugation strategy	mechanism	advantages	limitations	specificity	bond stability	simplicity	biocompatibility	scalability	applications in liver disease	key studies
maleimide–thiol coupling	covalent bond between maleimide-functionalized lipids and thiol groups (e.g., cysteine residues)	high stability, site-specificity, mild reaction conditions, suitable for PEGylated systems	requires thiol-modified peptides; maleimide hydrolysis at high pH	●●●	●●	●●	●●●	●●	liver fibrosis (aHSCs), HCC targeting via cRGD and GE11 peptides	^20,44,47,50^
NHS–ester amide bond	covalent bond formation between NHS-activated lipids and peptide amine groups	simple chemistry, mild aqueous conditions, preserves bioactivity	hydrolytic instability of NHS esters; nonspecific with multiple amines	●●	●●	●●●	●●●	●●	HCC targeting via CPP; stable peptide-lipid conjugates with preserved cell penetration	^78^
click chemistry (SPAAC)	bio-orthogonal azide–cyclooctyne cycloaddition forming stable triazole bonds	catalyst-free, site-specific, high efficiency, stable under biological conditions	complex synthetic requirements; limited commercial availability	●●●	●●●	●	●●●	●	ASGR1-targeted delivery via stem cell-derived vesicles; adaptable to liposomes	^94^
EDC/NHS carbodiimide coupling	amide bond between activated carboxyl (via EDC/NHS) and amine on lipid or peptide	no need for peptide modification (if carboxylic termini present), water-compatible	uncontrolled conjugation, less site-selective	●	●●	●●	●●●	●●	glycyrrhetinic acid-modified liposomes for curcumin and CA4P delivery in HCC	^91^
biotin–streptavidin linkage	strong noncovalent binding between biotinylated peptides and streptavidin-coated liposomes	ultrahigh affinity, reversible, modular screening possible	immunogenicity of streptavidin; *in vivo* instability	●●	●	●●●	●	●●	HSPG-targeting for liver cancer; potential for modular targeting system design	^93^
disulfide bond (SPDP linker)	reversible redox-sensitive disulfide linkage between SPDP-modified liposomes and thiol-containing peptides	enables release under reductive tumor conditions; compatible with complex liposomal systems	sensitive to extracellular reducing agents; less stable than thioether	●●	●	●●	●●	●●	EGFR-targeted magnetic polymeric liposomes (MPLs) for HCC using SPDP and external magnetic guidance	^61^

a Symbol-based scoring is used to facilitate rapid comparison of conjugation strategies across key translational parameters. ●●● = High/Favorable. ●● = Moderate. ● = Low/Challenging.

The scores in Table 3 were derived from a synthesis of published experimental data, established chemical principles, and peer-reviewed studies cited in Section 2.3. For instance, maleimide–thiol coupling and click chemistry (SPAAC) were ranked as Easy or Moderate in most categories due to their site-selective covalent linkages and well-documented success in targeted nanomedicine (e.g., Du et al.; Lu et al.). In contrast, NHS–ester and EDC/NHS systems, though widely used, received lower marks in specificity due to hydrolytic instability and potential for uncontrolled multisite conjugation (Ding et al.; Jiang et al.). Simplicity was assessed based on factors like reagent availability, aqueous vs organic compatibility, need for catalysts, and sensitivity to pH. Biocompatibility rankings reflect the immunogenic risk of systems such as biotin–streptavidin and the toxicity concerns associated with certain byproducts (e.g., copper in traditional click chemistry). Scalability considers the compatibility of each method with industrial translation, including use in microfluidics, thin-film hydration, or cGMP-compliant production systems (Tang et al.; Wu et al.).

This semiquantitative scoring framework is not intended to be absolute but rather serves as a practical decision-making aid for selecting optimal peptide conjugation methods in the design of liver-targeted liposomal formulations.

## 3 Liposomal Therapy: Passive Accumulation vs Active Targeting

Lipid nanoparticles (LNPs) encounter several biological barriers upon systemic administration that limit their efficient delivery to liver cells. Following intravenous injection, LNPs can interact nonspecifically with serum proteins, including immunoglobulins and complement proteins, leading to opsonization, aggregation, and rapid clearance through the RES by KCs and liver sinusoidal endothelial cells (SECs).^27,95^ To address this, stealth liposomes coated with PEG have been developed to avoid recognition by plasma proteins and prevent early clearance by the RES, improving pharmacokinetics and biodistribution.^96^

Liver-targeted delivery strategies are broadly categorized into passive and active targeting approaches.^95^ Passive targeting takes advantage of the liver’s unique anatomy and physiology, particularly its fenestrated vasculature and high-RES activity. Nanoparticles of appropriate size (typically below 200 nm and ideally under 100 nm) can traverse sinusoidal fenestrations and accumulate in hepatic tissue. While RES-mediated uptake is often considered a limitation for systemic delivery due to rapid clearance, it can be beneficial when therapeutic action is desired within RES-active regions such as fibrotic or inflamed liver tissue. However, excessive uptake by KCs may restrict bioavailability to nonphagocytic liver cells, such as hepatocytes or HCC cells.^97,98^

In contrast, active targeting involves the functionalization of liposomes with specific ligands such as proteins, antibodies, peptides, or carbohydrates that recognize and bind to disease-associated receptors in liver cells. This strategy enhances selectivity and cellular uptake through receptor-mediated endocytosis, improving therapeutic precision while minimizing off-target effects.^7^

In this review, we focus primarily on active targeting via peptide conjugation, highlighting how the rational design of peptide-functionalized liposomes can overcome biological barriers and achieve selective drug delivery in liver fibrosis and HCC.

### 3.1 Peptide-Targeted Liposomes for Selective Drug Delivery

Peptide-functionalized liposomes offer an effective strategy for achieving selective drug delivery in complex liver diseases, including liver fibrosis and HCC.^32^ By attaching targeting peptides to the liposomal surface, these systems exploit specific receptor–ligand interactions to enhance cellular uptake while reducing off-target effects.^99^ In addition, the use of well-characterized peptides provides regulatory advantages, as peptides are chemically defined and generally less immunogenic than full-length antibodies.^100^

In liver-directed therapies, four major receptor families have emerged as dominant targets for peptide-liposome conjugates: integrins, Human TfRs, growth factor receptors, and G protein-coupled receptors (GPCRs).^101^ Among these, integrins, particularly αvβ3 and αvβ5, are notably overexpressed on aHSCs in fibrotic livers and on the tumor vasculature in HCC.^101,102^ An overview of the major receptor families targeted by peptide-functionalized liposomes in liver diseases is provided in Figure 4. The figure serves as a comparative summary of integrins, TfR, HER-family receptors, and GPCRs, highlighting their relative disease specificity, expression patterns, internalization behavior, and clinical translational potential. The following subsections build upon this framework by discussing each receptor class in detail.

**Figure 4:**
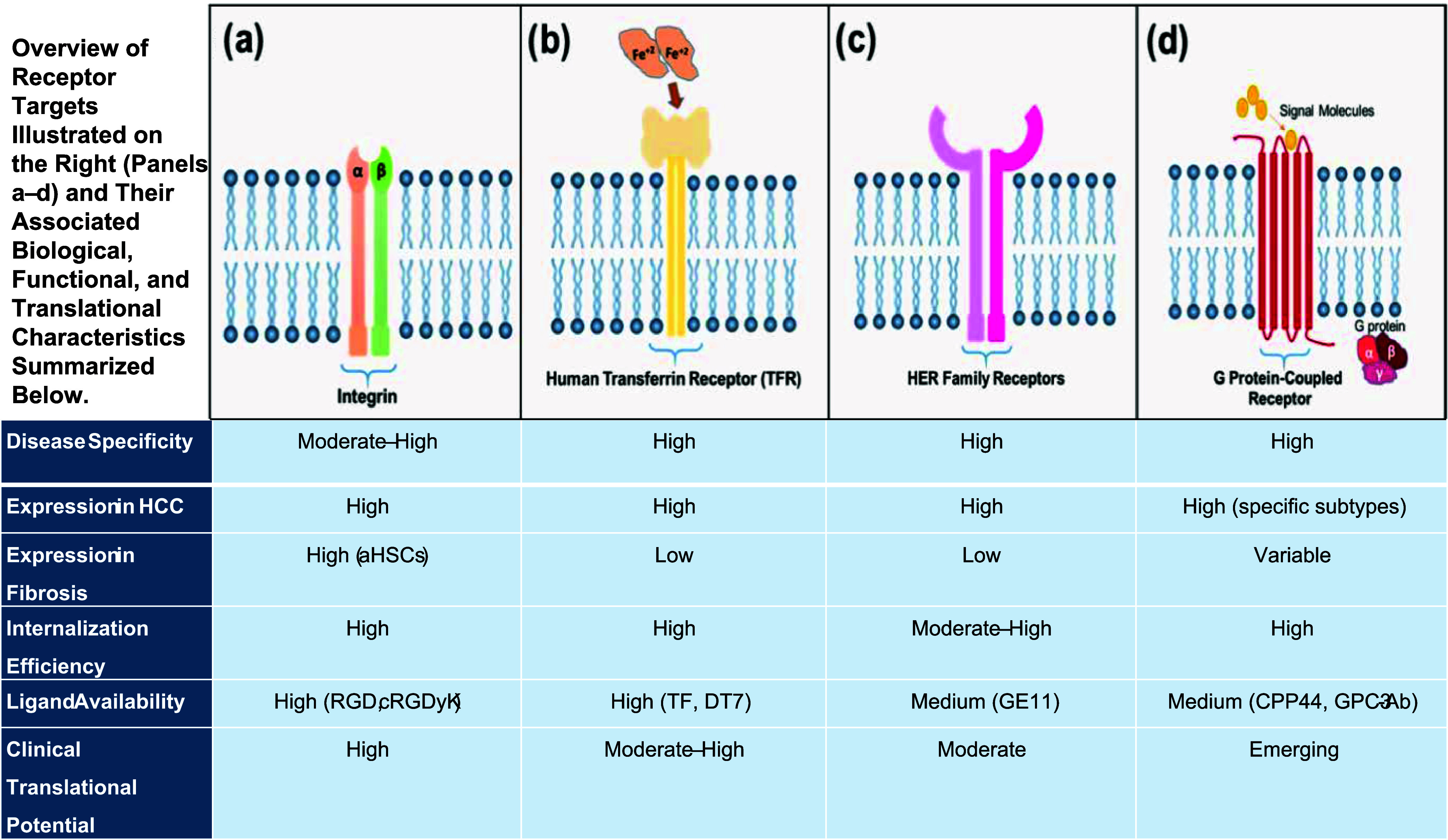
Major cell-surface receptors overexpressed in liver diseases and hepatocellular carcinoma: (a) integrins (α/β heterodimers) involved in cell adhesion and extracellular matrix interactions, (b) human transferrin receptor (TfR) mediating iron uptake through transferrin binding, (c) HER-family receptor tyrosine kinases implicated in tumor growth and proliferation, and (d) G protein–coupled receptors (GPCRs) involved in ligand-induced intracellular signaling. The table summarizes disease specificity, expression in hepatocellular carcinoma and liver fibrosis, internalization efficiency, ligand availability, and clinical translational potential.

Human transferrin receptors are single-chain transmembrane glycoproteins involved in cellular iron uptake.^18^ Transferrin binds ferric ions and enters cells through receptor-mediated endocytosis via the TfR. Because rapidly proliferating cancer cells have increased iron requirements, many tumor cell lines, including HepG2 and MDA-MB-231, exhibit elevated levels of TfR expression. This overexpression can be exploited to enhance the targeted delivery of therapeutic payloads using TfR-directed liposomal systems.^18^

Another important receptor class for liver cancer targeting is EGFR, also known as HER1, a receptor tyrosine kinase frequently overexpressed in HCC.^103^ Short peptide ligands, such as GE11, selectively bind EGFR without activating downstream signaling pathways. This enables receptor-mediated endocytosis of liposomal cargo while avoiding undesired proliferative signaling.^17^ This strategy has facilitated tumor-specific delivery while minimizing undesired proliferative signaling associated with ligand engagement.

In addition to integrins and receptor tyrosine kinases, GPCRs have gained attention as targets for peptide-liposome conjugation, particularly in liver diseases involving inflammatory or endocrine pathways.^104^ Chemokine receptors, such as CXCR4, and hormonal receptors, including the angiotensin II type 1 receptor, have been successfully targeted using peptide ligands.^105^ For instance, targeting CXCR4 in HCC has been associated with inhibition of metastatic pathways and enhanced drug accumulation in tumor tissues.^106^ Similarly, somatostatin receptor subtypes (SSTRs), overexpressed in neuroendocrine liver tumors, present an opportunity for peptide-directed delivery using somatostatin analogs.^107^

Collectively, these receptor families form the foundation of peptide-guided liposomal delivery strategies for liver therapeutics. Their differential expression in fibrotic and malignant hepatic tissues enables a level of selectivity that is difficult to achieve with nontargeted formulations.^108^ As the field progresses, a deeper understanding of receptor expression patterns, internalization mechanisms, and downstream signaling effects will be essential for optimizing therapeutic performance. The following sections examine each receptor class in greater detail, highlighting the design and efficacy of peptide–liposome systems in both *in vitro* and *in vivo* liver models and discussing their translational potential.

The sections that follow examine each receptor class in depth, exploring the design and performance of peptide-liposome constructs in both *in vitro* and *in vivo* liver models and drawing translational insights for the development of next-generation nanomedicines.

#### 3.1.1 Integrin Receptors

Recent advances in targeted nanomedicine have highlighted integrins as crucial receptors for precision drug delivery in liver pathologies. These heterodimeric cell adhesion molecules, particularly the αvβ3 and α5β1 subtypes, are significantly overexpressed in both HCC and aHSCs during fibrogenesis.^109^ In HCC, the upregulation of αvβ3 integrin correlates strongly with tumor aggressiveness and metastatic potential.^16^ The distinctive RGD (Arg-Gly-Asp) tripeptide sequence present in many extracellular matrix proteins has become a focal point for developing integrin-specific targeting strategies, as it demonstrates high binding affinity to multiple integrin subtypes.^19^

Du et al. pioneered integrin-targeted liposomal therapy for liver fibrosis by using cRGD-modified sterically stable liposomes (cRGD-SSL) to deliver IFN-α1b to aHSCs, achieving a 10-fold increase in liver accumulation and significant fibrosis reduction.^47^ Building on this, Li et al. used a similar cRGD-based strategy with PEGylated liposomes encapsulating hepatocyte growth factor (HGF), prepared via thin-film hydration. Their formulation also demonstrated enhanced liver targeting, reduced fibrosis markers, and prolonged HGF circulation. Together, these studies validate cRGD–integrin targeting as an effective approach for antifibrotic nanotherapy.^50^

Liu et al. introduced a more refined and disease-specific approach by functionalizing sterically stable liposomes with the cyclic peptide cRGDyK to target αvβ3 integrins selectively overexpressed on aHSCs. These liposomes encapsulated vismodegib, a hedgehog pathway inhibitor, and effectively suppressed HSC activation, attenuating fibrosis in both BDL and thioacetamide-induced injury models. Notably, this system displayed exceptional cell selectivity, avoiding uptake by quiescent HSCs and hepatocytes, thereby improving therapeutic precision over earlier systems.^110^

However, heterogeneous integrin expression and impaired cellular internalization often limit the therapeutic reach of liposomal systems in tumors. To address this, Amin et al. designed a dual-ligand liposome modified with both an RGD peptide, targeting tumor vasculature via αv integrins, and a TAT peptide, derived from HIV-1 transactivator of transcription, to promote extravascular tumor cell penetration. Intravital imaging revealed that while RGD-modified liposomes showed variable vascular and parenchymal localization, TAT-modified liposomes exhibited consistent extravascular targeting. The dual-ligand system combined the strengths of both, demonstrating improved vascular binding and a deeper tumor penetration. When loaded with doxorubicin, these dual-modified liposomes showed enhanced therapeutic efficacy compared to single-ligand formulations, despite a slightly increased clearance rate.^55^

Together, these studies illustrate the evolution and growing complexity of peptide-functionalized liposomes for liver-targeted therapy. Early RGD-based systems pioneered the strategy of targeting αvβ3 integrins on aHSCs, and later studies refined ligand specificity, expanded receptor targets, and integrated dual-targeting schemes to address both fibrosis and HCC. These advancements mark a shift from proof-of-concept toward translationally relevant nanomedicines with potential for clinical application in liver disease management.

Despite these promising advances, research in this area remains relatively limited to a handful of integrin subtypes. Most studies focus on αvβ3 due to its established role in angiogenesis and cancer cell invasion. However, integrins such as α5β1 and αvβ5, which are also upregulated in liver tumors and their vasculature, remain underexplored in liposomal delivery systems.

#### 3.1.2 Human Transferrin Receptors (TfRs)

The human TfR, an iron-regulating transmembrane glycoprotein overexpressed in many solid tumors including HCC, has become an attractive target for tumor-selective nanomedicine.^18^ To exploit this overexpression, Tang et al. designed a stabilized retro-inverso peptide, DT7, as a transferrin-mimetic ligand with enhanced proteolytic stability and binding affinity to TfR.^10^ Compared to native LT7 and its transferrin-conjugated counterparts, DT7-conjugated liposomes (DT7-LIP) exhibited significantly improved tumor accumulation, cellular uptake, and therapeutic efficacy in HCC models, particularly when loaded with docetaxel (DTX). The enhanced targeting capability and resistance to enzymatic degradation highlighted the value of DT7 as a robust peptide ligand for liposomal drug delivery to TfR-positive tumors.^60^ Building upon this strategy, Zhao et al. developed a transferrin-modified liposomal formulation of triptolide (TF-TP@LIP) using the thin-film hydration method. These PEGylated liposomes achieved high drug encapsulation, serum stability, and preferential tumor accumulation in HepG2-bearing nude mice. Notably, TF-TP@LIP significantly improved cellular uptake and reduced the IC_50_ of triptolide compared to nontargeted liposomes, while also minimizing off-target toxicity in major organs. Together, these studies confirm that TfR-targeted liposomes, whether functionalized with retro-inverso peptides or native transferrin, offer a promising platform for enhancing the specificity, efficacy, and safety of chemotherapeutics in liver cancer treatment.^18^

Building on the therapeutic promise of TfR-mediated targeting, Yang et al. developed transferrin-conjugated, erianin-loaded liposomes (Tf-LP-ERN) using the ethanol injection method to overcome the solubility and off-target limitations of the natural compound erianin.^70^ The resulting nanoparticles exhibited optimal physicochemical properties with a uniform particle size (∼88 nm) and enhanced serum stability. *In vitro* studies in HepG2 and SMMC-7721 liver cancer cells demonstrated superior cellular uptake, mitochondrial disruption, and apoptosis induction by Tf-LP-ERN compared to nontargeted ERN formulations. *In vivo*, Tf-LP-ERN achieved enhanced tumor accumulation and significantly inhibited tumor growth in xenograft mouse models while preserving body weight and organ morphology. Mechanistically, Tf-LP-ERN modulated oxidative stress pathways—upregulating pro-apoptotic proteins (Bax, Bad, PUMA) and antioxidant markers (Nrf2, HO-1, SODs), while downregulating antiapoptotic (Bcl-2) and pro-inflammatory (P-NF-κB, P-IKKα/β) signaling. These findings reinforce the potential of transferrin-functionalized liposomes as a tumor-selective delivery platform, combining targeted delivery with immunomodulatory and apoptotic mechanisms for liver cancer therapy.

In a parallel line of development, Yu et al. explored transferrin receptor-targeting through peptide–drug conjugates (PDCs), offering a complementary strategy to liposomal delivery.^53^ They synthesized LT7-SS-DOX and its retro-inverso analogue DT7-SS-DOX using a redox-sensitive disulfide linker. Both conjugates selectively killed TfR-overexpressing tumor cells while sparing normal cells, with DT7-SS-DOX showing greater serum stability and controlled release. Although nonliposomal, this work highlights the strong targeting fidelity of TfR-binding peptides and their potential integration into future liposomal systems for HCC therapy.

#### 3.1.3 HER-Family Receptors

Epidermal growth factor receptor (EGFR/HER1), a member of the HER receptor tyrosine kinase family, is frequently overexpressed in HCC, making it an attractive target for peptide-mediated drug delivery systems.^111^ Over the past decade, significant progress has been made in the development of EGFR-targeted liposomal systems functionalized with peptides, with a particular emphasis on improving tumor specificity and overcoming delivery barriers in HCC. One such effort was conducted by Tang et al., who examined the intratumoral behavior of GE11 peptide-functionalized PEGylated liposomes (GE11-TLs) in an EGFR-overexpressing SMMC-7721 HCC xenograft model. Through laser scanning confocal microscopy and immunohistochemistry, the study revealed that although GE11-TLs demonstrated specific EGFR binding, their deep tissue penetration was hampered by biological obstacles such as dense collagen networks, a high macrophage presence, and heterogeneous tumor vascularization. Importantly, the study identified the ″binding-site barrier″, a phenomenon wherein nanoparticles rapidly bind to EGFR-positive cells near vasculature as the primary limitation to uniform distribution. These findings underscore the need to fine-tune peptide density and nanoparticle architecture to bypass receptor saturation and enhance intratumoral penetration.^112^

Complementing this approach, Lin et al. developed a multifunctional EGFR peptide-conjugated magnetic polymeric liposome (MPL) platform for targeted liver cancer therapy. These PEG-coated MPLs were composed of octadecyl quaternized carboxymethyl chitosan (OQC), PEGylated OQC, cholesterol, and embedded superparamagnetic iron oxide nanoparticles, enabling dual targeting through both receptor-mediated recognition and external magnetic field guidance. Paclitaxel-loaded MPLs achieved high EE (>90%) and maintained a uniform particle size (∼102 nm). *In vivo*, the system demonstrated superior tumor accumulation and therapeutic response in a subcutaneous HCC mouse model, confirming the synergistic benefit of combining EGFR-specific ligand targeting with physical delivery enhancement.^72^ Collectively, these studies highlight the evolution of EGFR-directed nanomedicine in HCC, emphasizing the importance of integrated strategies that address both molecular and physiological barriers to optimize the therapeutic efficacy.

#### 3.1.4 G Protein-Coupled Receptors (GPCRs)

The growing interest in receptor-targeted nanomedicine has expanded to include GPCRs—a vast and pharmacologically rich receptor family with emerging relevance in liver cancer and fibrosis.^113^ GPCRs are particularly attractive targets for peptide-functionalized liposomal systems due to their high expression on diseased liver tissues, strong internalization upon ligand binding, and active involvement in processes such as tumor growth, metastasis, angiogenesis, and fibrogenesis.^114^

Lin et al. developed a dual-ligand liposomal formulation for the targeted delivery of arsenic trioxide (ATO) in HCC, leveraging both receptor specificity and enhanced cellular penetration.^21^ Their system incorporated two targeting moieties: an anti-GPC3 antibody for the active recognition of glypican-3 (GPC3), a membrane-bound proteoglycan overexpressed in HCC, and CPP44, a cell-penetrating peptide known to facilitate intracellular uptake in liver tumor cells. The liposomes, loaded with ATO, were initially guided to the tumor site through a combination of passive accumulation (via the EPR effect) and GPC3-mediated active targeting. CPP44 further enhanced intracellular delivery, overcoming the membrane transport barriers. *In vivo* studies in HCC-bearing mice demonstrated a tumor inhibition rate of 63.43%, outperforming single-ligand or nontargeted controls. This work highlights the synergistic potential of combining antibody-based targeting with CPP-mediated delivery in a single nanocarrier, offering a versatile strategy for improving the therapeutic index of potent but systemically toxic agents like ATO.^21^

Table 4 summarizes key preclinical studies that have employed receptor-targeted liposomal formulations in liver pathologies, such as fibrosis and HCC. The table outlines the target receptor, conjugation strategy, liposome composition, encapsulated therapeutic payload, disease model, and major outcomes, offering a comparative view of how each system contributes to improved delivery efficiency and therapeutic performance.

**Table 4:** Overview of Peptide- and Ligand-Functionalized Liposomal Systems Targeting Liver-Relevant Receptors

receptor	study	target/strategy	liposome composition	therapeutic payload	disease model	key outcomes
integrin	Du et al. (2007)	cRGD-targeted delivery to aHSCs (αvβ3 integrins)	EPC/Chol/mPEG-DOPE/MAL-PEG-DOPE	IFN-α1b	liver fibrosis (BDL rat model)	10× higher liver accumulation, reduced fibrosis markers
	Li et al. (2008)	cRGD-targeted delivery of HGF	EPC/Chol/mPEG-DOPE/MAL-PEG-DOPE	HGF	liver fibrosis	improved hepatic delivery, reduced α-SMA and collagen, prolonged circulation
	Liu et al. (2019)	cRGDyK targeting αvβ3 on activated HSCs	sterically stable liposomes with cRGDyK-DSPE-PEG modification	vismodegib	fibrosis (BDL and TAA-induced injury models)	HSC-specific targeting, suppressed activation, minimal off-target uptake
	Amin et al. (2021)	dual-ligand system (RGD for vasculature + TAT for tumor cell penetration)	liposomes with RGD and TAT peptide modifications	doxorubicin	solid tumors (HCC-relevant)	improved vascular/extravascular delivery, enhanced efficacy, mild clearance boost
human transferrin receptors (TfRs)	Tang et al. (2019)	TfR-targeted delivery via retro-inverso DT7 peptide	DT7-modified PEGylated liposomes	docetaxel	HCC xenograft models	enhanced stability, uptake, and tumor accumulation; improved efficacy over LT7 and transferrin control
	Zhao et al. (2023)	native transferrin-conjugated PEGylated liposomes	TF-PEG-liposomes (via thin-film hydration)	triptolide	HepG2-bearing nude mice	improved IC_50_, tumor accumulation, and safety profile; minimized off-target toxicity
	Yang et al. (2021)	transferrin-conjugated PEGylated liposomes via ethanol injection	Tf-LP-ERN (PEG-liposomes + ERN + transferrin)	erianin	HepG2 & SMMC-7721 xenograft models	enhanced oxidative stress modulation and apoptosis; reduced inflammation and tumor growth
	Yu et al. (2024)	TfR-targeted PDC using retro-inverso DT7 peptide (nonliposomal)	DOX conjugated to DT7 or LT7 via disulfide linker	doxorubicin (PDCs)	TfR-overexpressing tumor cells	DT7-DOX conjugates showed higher serum stability, selective cytotoxicity, and better *in vitro* efficacy
HER-family receptors	Tang et al. (2020)	GE11-mediated EGFR-targeted delivery; analysis of tumor penetration barriers	PEGylated liposomes modified with GE11 peptide	not specified (focus on biodistribution)	HCC (SMMC-7721 xenograft)	EGFR-specific binding; poor deep penetration due to receptor-binding site barrier, dense collagen, and macrophage infiltration
	Lin et al. (2020)	EGFR peptide + magnetic targeting for dual-mode accumulation	OQC/PEG-OQC/Chol + superparamagnetic iron oxide nanoparticles, PEG-EGFR peptide-modified	paclitaxel	subcutaneous HCC mouse model	high encapsulation (>90%); enhanced tumor uptake via EGFR + magnetic force; improved antitumor efficacy
G protein-coupled receptors	Lin et al. (2025)	dual-ligand targeting: anti-GPC3 antibody (tumor-specific) + CPP44 (cell penetration)	ATO-loaded PEGylated liposomes with anti-GPC3 & CPP44	arsenic trioxide (ATO)	HCC-bearing mouse model	tumor inhibition rate of 63.4%; enhanced targeting and uptake vs single-ligand or untargeted controls

## 4 Clinical Development Landscape and Translational Barriers of Peptide-Modified Liposomes for Hepatic Therapy

Peptide-functionalized liposomes offer significant promise for targeted therapy in liver fibrosis and HCC, yet their clinical translation remains fraught with numerous challenges. Despite encouraging preclinical data, no peptide-modified liposomal formulation has yet received FDA approval for liver disease, highlighting the complexity of bridging laboratory success with clinical application.^115^

A central barrier is the limited therapeutic efficacy of many targeted systems relative to that of standard treatments. Although some formulations show enhanced tumor or tissue uptake in preclinical models, these improvements frequently do not translate into superior patient outcomes. The thermosensitive liposomal doxorubicin ThermoDox, tested in combination with local ablation for HCC, reached Phase III evaluation but did not achieve its primary clinical endpoints, illustrating the gap between localized release and meaningful survival benefits.^116^ The HERMIONE phase II/III trial (NCT02213744) evaluated the efficacy of the addition of MM-302 to trastuzumab for metastatic HER2-positive breast cancer. Unfortunately, the study was prematurely terminated because it was found not to confer any benefit when compared with the control.^117^

Several ligand-targeted liposomal and immunoliposomal platforms have progressed into early-phase clinical evaluation, offering valuable insights into both the promise and persistent limitations of targeted nanocarrier translation. Examples include C225-ILs-DOX, SGT-53, and SGT-94, which have entered Phase I or II trials primarily in solid tumors outside the liver.^117^ These systems demonstrated acceptable safety profiles, confirmed target engagement, and in some cases disease stabilization or partial responses, supporting the feasibility of ligand-mediated delivery in humans. However, despite encouraging preclinical performance and early clinical signals, none of these platforms has yet achieved regulatory approval or widespread clinical adoption.

In contrast, several peptide-based therapeutics such as Lutathera (Novartis) for gastroenteropancreatic neuroendocrine tumors and Pepaxto (Oncopeptides) for multiple myeloma have successfully gained FDA approval; notably, these agents do not employ peptide-functionalized liposomal delivery systems and are not indicated for liver diseases. Together, these outcomes highlight a persistent translational gap specific to peptide-modified liposomal nanocarriers, in which demonstration of safety, target engagement, and enhanced delivery does not necessarily translate to durable clinical benefit. The clinical experiences of both liposomal and nonliposomal peptide-based therapies therefore, provide important context for hepatic applications, where disease heterogeneity and biological barriers further complicate translation.

### 4.1 Translational Barriers in Peptide-Functionalized Liposomal Delivery for Liver Disease

#### 4.1.1 Receptor Heterogeneity and Delivery Barriers

One major obstacle is the heterogeneity of the receptor expression within diseased liver tissue. Activated HSCs in fibrotic regions and malignant hepatocytes in HCC exhibit divergent receptor profiles, such as upregulation of integrin αvβ3, which may vary both spatially and temporally within fibrotic septa or hypoxic tumor zones.^109^ This variability can result in uneven liposome binding and drug delivery, potentially leaving poorly perfused or receptor-deficient regions untreated. In addition, the extracellular matrix (ECM) in liver fibrosis and HCC poses significant physical barriers to nanoparticle penetration.^118^ Dense collagen networks in fibrotic tissue and desmoplastic stroma in tumors can obstruct liposome access, necessitating careful optimization of particle size, surface charge, and ligand multivalency, and possibly the coadministration of ECM-degrading enzymes.^119^

#### 4.1.2 Pharmacokinetics and Immune Interaction

Pharmacokinetic and biodistribution challenges further complicate the therapeutic performance of peptide-functionalized liposomal systems.^32^ PEGylation is widely employed to prolong systemic circulation by reducing recognition and clearance by the mononuclear phagocyte system (MPS); however, its effects are highly context dependent. In liver-targeted applications, partial uptake by resident macrophages such as KCs may facilitate passive hepatic accumulation, particularly in fibrotic or tumor-bearing livers.^120^ Nevertheless, PEGylation does not fully abrogate immune recognition and may, in some cases, reduce beneficial RES-mediated uptake depending on the therapeutic objective.^121^

Importantly, repeated administration of PEGylated liposomes can trigger immune responses, including the generation of anti-PEG antibodies, leading to the accelerated blood clearance (ABC) phenomenon and reduced circulation time upon subsequent dosing.^122^ In addition, both the liposomal surface and conjugated peptides may activate the complement system, resulting in opsonization, enhanced macrophage uptake, and potential infusion-related reactions.^123^ Such complement activation–related pseudoallergy (CARPA) has been reported for several nanoparticle-based therapeutics and represents a critical barrier to clinical translation.^123^

The density and presentation of peptide ligands on the liposome surface further modulate immune interactions. High ligand density can promote opsonization and immune clearance, whereas insufficient density compromises receptor-mediated targeting efficiency.^99^ Off-target accumulation in liver sinusoidal endothelial cells and KCs remains a recurring issue, potentially diluting delivery to fibrotic niches or tumor nodules and reducing therapeutic precision.^7^ To mitigate these immune-related challenges, several strategies have been proposed, including the use of alternative stealth coatings (e.g., zwitterionic polymers or PEG analogues), optimization of PEG chain length and architecture, peptide masking or cleavable shielding approaches, and careful control of ligand density and dosing regimens. Collectively, these considerations highlight the need to balance prolonged circulation, immune compatibility, and effective targeting when designing peptide-functionalized liposomal systems for liver-directed therapy.^124^

The *in vivo* stability of peptide–liposome conjugation is another critical determinant of targeting efficacy and pharmacokinetic performance.^125^ Peptides are commonly attached to liposomal membranes through covalent linkages involving lipid anchors, such as amide bonds formed via EDC/NHS coupling, thioether bonds generated by maleimide–thiol chemistry, or disulfide linkages designed to be redox-responsive.^126^ Among these, stable amide and thioether bonds generally exhibit high resistance to hydrolysis and enzymatic degradation under physiological conditions, thereby preserving peptide attachment during systemic circulation.^127,128^ In contrast, disulfide bonds and other stimuli-responsive linkers may undergo cleavage in the reductive intracellular environment or in pathological tissues with elevated glutathione levels, which can be advantageous for controlled ligand shedding or intracellular release but may reduce surface stability during circulation.^129^

*In vivo*, additional factors such as shear stress, serum protein adsorption, enzymatic activity, and immune interactions can influence linker integrity and peptide retention on the liposomal surface.^127^ Premature detachment of targeting peptides may diminish receptor-mediated uptake and compromise targeting specificity, whereas excessively stable linkages may increase immune recognition or hinder intracellular processing.^130^ Consequently, linker chemistry must be carefully selected to balance systemic stability with functional responsiveness.^131^ Strategies such as spacer optimization (e.g., PEG length), steric shielding, and the use of cleavable versus noncleavable linkers are increasingly employed to fine-tune peptide retention and biological performance *in vivo*.^132^

#### 4.1.3 Drug Resistance and Ligand Desensitization

Resistance mechanisms within liver tumors add another layer of complexity. HCC often overexpresses ATP-binding cassette (ABC) transporters such as P-glycoprotein and MRP2, which can actively efflux chemotherapeutic agents from the cell interior even after successful liposomal uptake.^133^ Although liposomes can circumvent some efflux via endosomal pathways, multidrug resistance remains a formidable barrier and may require combination strategies with efflux pump inhibitors or the use of drugs less prone to extrusion.^134^ Additionally, chronic stimulation of target receptors by peptide ligands can lead to receptor internalization and downregulation, thereby decreasing liposome uptake in subsequent dosing cycles.^135^ Optimal dosing regimens, ligand density, and payload selection must be considered to mitigate receptor desensitization.

#### 4.1.4 Manufacturing and Regulatory Hurdles

The path to clinical applications also faces regulatory and manufacturing challenges. FDA approval mandates robust demonstration of consistent liposome synthesis, stability, safety, and efficacy, often through multiphase clinical trials.^136^ The incorporation of peptide ligands adds complexity in terms of chemical conjugation, reproducibility, and batch-to-batch consistency.^137^ Scaling up laboratory techniques like microfluidic mixing or extrusion to industrial levels must be achieved without altering key parameters such as liposome size distribution, peptide density, and EE.^138^ Ensuring sterility and effectively removing unbound peptide or free drug further complicates production.^138^

#### 4.1.5 Patient Stratification and Companion Diagnostics

On the clinical front, the success of peptide-targeted liposomes depends heavily on patient stratification. Given the variability in receptor expression, identifying individuals whose fibrotic or tumor tissues overexpress the intended target is essential.^139^ Companion diagnostic tools such as imaging with radiolabeled peptides may become necessary to select suitable candidates, although this introduces additional cost and complexity.^139^

## 5 Future Directions and Emerging Trends in Liposomal Drug Delivery Systems for Liver Diseases

Recent advances in peptide-functionalized liposomal systems have extended their utility beyond classical chemotherapy into multifunctional platforms that integrate diagnostics, gene therapy, immune modulation, and regenerative medicine. These next-generation formulations are increasingly being designed to address the complexities of HCC and liver fibrosis by combining precise targeting, stimuli-responsiveness, and payload versatility.

### 5.1 Multifunctional and Theranostic Liposomes

Several studies have demonstrated that liposomes can be coengineered to deliver both therapeutic and imaging agents in a single platform, enabling real-time tracking and treatment assessment. For example, Li et al. developed a multifunctional theranostic liposomal system incorporating the tumor-penetrating peptide iRGD, the chemotherapeutic agent 10-hydroxycamptothecin, and the imaging dye indocyanine green. This system enabled photoacoustic and ultrasound dual-modality imaging in HCC models while also allowing low-intensity focused ultrasound-triggered drug release at tumor sites. *In vitro* and *in vivo* studies demonstrated enhanced tumor targeting, deep tissue penetration, and potent pro-apoptotic activity following ultrasound activation. This approach underscores the potential of peptide-guided, image-responsive liposomes as precision theranostic platforms for real-time monitoring and tailored treatment of HCC.^140^

### 5.2 Gene and RNA-Based Therapeutics

Targeted delivery of genetic cargo using peptide-functionalized liposomes has gained traction, particularly for liver fibrosis, where the modulation of gene expression is essential. Ullah et al. developed CXCR4-targeted combination liposomes (CTC liposomes) for the codelivery of pirfenidone (PF) and AMD3100, aiming to treat TGFβ-induced activation of HSC-T6 cells, a key driver of liver fibrosis. The liposomes were prepared via thin-film hydration and demonstrated high drug encapsulation, CXCR4-specific uptake, and effective caveolae-mediated internalization. Compared to free drugs, CTC liposomes showed superior antifibrotic efficacy, significantly downregulating fibrogenic markers, including α-SMA, CXCR4, TGFβ, and P-p38, and inducing 87.3% apoptosis in activated HSCs. The *in vivo* imaging and biodistribution confirmed targeted accumulation in fibrotic liver tissue. These findings highlight CXCR4 as a promising GPCR target and support the potential of dual-agent, receptor-targeted liposomes in reversing liver fibrosis.^141^

### 5.3 Dual-Targeting Strategies for Heterogeneous Tissues

To overcome receptor heterogeneity within the liver microenvironment, researchers have explored dual-targeting strategies that combine two ligands for improved selectivity and uptake. Lin et al. designed a dual-ligand liposome modified with an anti-GPC3 antibody and CPP44 peptide to deliver arsenic trioxide in HCC. This system exploited GPC3-mediated tumor targeting and CPP-mediated membrane translocation, achieving a 63% tumor inhibition rate *in vivo*. Such combinatorial designs improve cell-type specificity, enhance intertumoral penetration, and offer a promising route for addressing variable receptor expression in both fibrosis and cancer.^56^

### 5.4 Stimuli-Responsive and Environment-Activated Systems

Peptide-functionalized liposomes are also being engineered to respond to local microenvironmental cues, such as low pH, high glutathione levels, or matrix metalloproteinase (MMP) activity. Cheng et al. developed hepatocyte-targeted, MMP-2-responsive liposomes (PPP-LIP) encapsulating 10-hydroxycamptothecin (HCPT) for enhanced liver tumor therapy. These liposomes were functionalized with myrcludex B to ensure hepatocyte-specific delivery and incorporated an MMP-2-cleavable PEG-TATp construct that exposed a cell-penetrating peptide in the tumor microenvironment. This dual-targeting strategy enabled selective accumulation in hepatic tumors and triggered drug release and cellular uptake, specifically in MMP-2-overexpressing tissues. Compared to conventional HCPT injections, PPP-LIP demonstrated superior antitumor efficacy in both *in vitro* and *in vivo* liver cancer models. Environment-activated targeting mechanisms such as these offer promising avenues for precise, site-specific drug delivery in malignant hepatic tissues.^58^

### 5.5 Immunomodulatory Liposomes and Combination Therapies

Recent work has also focused on combining liposomal chemotherapy with immunotherapy. In one study, Amin et al. developed dual-ligand liposomes (RGD + TAT) to enhance both vascular targeting and tumor penetration, improving doxorubicin efficacy while promoting T-cell infiltration.^55^ Other designs have explored the codelivery of immunomodulators, such as anti-PD-L1 antibodies or small-molecule checkpoint inhibitors, alongside chemotherapeutics within peptide-targeted liposomes.^142^ These formulations aim to reverse tumor-induced immunosuppression and boost endogenous antitumor responses, which are particularly important in the immunologically cold environment of HCC.

### 5.6 Regenerative and Antifibrotic Liposome Platforms

A growing body of literature also explores regenerative approaches using liposomes. Lai et al. developed a vitamin A-functionalized fluorinated peptide-lipid hybrid nanoparticle for HSC-targeted chemo-gene therapy. These nanoparticles codeliver sorafenib, which promotes collagen degradation, and siRNA against HSP47, a key mediator of collagen synthesis. The dual-action approach effectively reduced extracellular matrix accumulation by simultaneously inhibiting collagen production and enhancing its breakdown. Vitamin A-mediated targeting enabled selective accumulation in activated HSCs via retinol-binding protein receptors, enhancing delivery efficiency. In fibrotic mouse models, this system restored liver function and attenuated fibrosis through reduced collagen deposition, a lower hydroxyproline content, and diminished fibrogenic marker expression. Such targeted nanoplatforms offer a compelling strategy for overcoming fibrotic barriers and remodeling liver tissue in advanced hepatic fibrosis.^143^ Additionally, liposomes functionalized with CXCR4-targeting peptides are being evaluated to recruit hepatic progenitor cells to sites of injury, thus supporting endogenous regeneration while delivering antifibrotic agents.^141^ These strategies emphasize a shift toward precision, multifunctionality, and combination therapies in peptide-functionalized liposomal nanomedicine for liver diseases.

## 6 Conclusion

Peptide-functionalized liposomal systems represent a versatile and increasingly sophisticated platform for precision therapy in liver fibrosis and HCC. By harnessing the unique biology of cell-surface receptors including integrins (αvβ3/αvβ5), HER-family RTKs (EGFR/HER1), and GPCRs (CXCR4, AT1R, SSTRs) researchers have demonstrated in preclinical models the ability to selectively deliver antifibrotic agents to activated HSCs and chemotherapeutics to malignant hepatocytes and the tumor microvasculature. Innovations such as cyclic RGD, GE11, iRGD–LyP-1 dual-ligand, and AT1R-binding peptides have yielded marked improvements in target cell uptake, tissue penetration, and therapeutic index compared to nontargeted liposomes.

Despite these advances, no peptide-modified liposomal formulation has yet achieved FDA approval, highlighting persistent challenges: receptor heterogeneity across fibrotic and tumor compartments, clearance by the mononuclear phagocyte system, multidrug resistance mechanisms, and the complexities of large-scale, reproducible manufacturing. Overcoming these barriers will require not only continued optimization of ligand affinity, liposome physicochemistry, and dosing regimens but also rigorous patient stratification using companion diagnostics to identify those most likely to benefit.

Looking ahead, the integration of next-generation peptides with stimuli-responsive linkers, multifunctional theranostic liposomes carrying imaging agents or gene-silencing payloads, and synergistic combination regimens with immunotherapies or regenerative factors promises to redefine liver-targeted nanomedicine. As these platforms transition from proof-of-concept toward clinical translation, they hold the potential to deliver truly personalized interventions that arrest fibrosis progression, eradicate tumor cells, and ultimately restore liver health. The path to the first FDA-approved peptide-targeted liposomal therapy for liver disease lies at the intersection of innovative chemistry, advanced biotechnology, and precision patient care.

## Abbreviations

α-SMA: α smooth muscle actin
α5β1: α5β1 integrin
αvβ3: αvβ3 integrin
αvβ5: αvβ5 integrin
ABC phenomenon: accelerated blood clearance phenomenon
ASGPR: asialoglycoprotein receptor
ASGR1: asialoglycoprotein receptor 1
AT1R: angiotensin II type 1 receptor
ATO: arsenic trioxide
Bad: Bcl-2-associated agonist of cell death
Bax: Bcl-2-associated X protein
BDL: bile duct ligation
CARPA: complement activation-related pseudoallergy
Chol: cholesterol
CPPs: cell-penetrating peptides
cRGD: cyclic arginine–glycine–aspartic acid peptide
cRGDyK: cyclic arginine–glycine–aspartic acid–tyrosine–lysine peptide
CTC liposomes: CXCR4-targeted combination liposomes
CXCR4: C–X–C chemokine receptor 4
DT7: retro-inverso transferrin receptor-targeting peptide
DTX: docetaxel
ECM: extracellular matrix
EDC: 1-ethyl-3-(3-(dimethylamino)­propyl)­carbodiimide
EE: encapsulation efficiency
EGFR: epidermal growth factor receptor
EPC: egg phosphatidylcholine
EPR: enhanced permeability and retention effect
ERN: erianin
FDA: Food and Drug Administration
GalNAc: N-acetylgalactosamine
GPCRs: G protein-coupled receptors
GPC3: glypican-3
GUVs: giant unilamellar vesicles
HCPT: 10-hydroxycamptothecin
HCC: hepatocellular carcinoma
HER1: human epidermal growth factor receptor 1
HER2: human epidermal growth factor receptor 2
HGF: hepatocyte growth factor
HIV-1: human immunodeficiency virus type 1
HO-1: heme oxygenase 1
HSCs: hepatic stellate cells
HSP47: heat shock protein 47
IC_50_: half maximal inhibitory concentration
IFN-α1b: interferon α-1b
iRGD: internalizing RGD peptide
KCs: Kupffer cells
LNPs: lipid nanoparticles
LP: liposome/liposomal formulation
LT7: L-peptide transferrin receptor-binding peptide
LUVs: large unilamellar vesicles
LyP-1: lymphatic homing peptide 1
M6PR: mannose-6-phosphate receptor
MAL: maleimide
MDA-MB-231: human breast cancer cell line
MLPs: magnetic polymeric liposomes
MLVs: multilamellar vesicles
MMP-2: matrix metalloproteinase 2
MMPs: matrix metalloproteinases
MRP2: multidrug resistance-associated protein 2
MPS: mononuclear phagocyte system
MVVs: multivesicular vesicles
NASH: nonalcoholic steatohepatitis
NF-κB: nuclear factor kappa B
NHS: *N*-hydroxysuccinimide
Nrf2: nuclear factor erythroid 2-related factor 2
OQC: octadecyl quaternized carboxymethyl chitosan
PD-L1: programmed death-ligand 1
PDGFR-β: platelet-derived growth factor receptor β
PDCs: peptide–drug conjugates
PEG: poly­(ethylene glycol)
PF: pirfenidone
PPP-LIP: MMP-2-responsive peptide–PEG–TAT liposomes
PUMA: p53 upregulated modulator of apoptosis
RES: reticuloendothelial system
RGD: arginine–glycine–aspartic acid peptide
RTKs: receptor tyrosine kinases
SECs: sinusoidal endothelial cells
siRNA: small interfering RNA
SMMC-7721: human hepatocellular carcinoma cell line
SODs: superoxide dismutases
SP94: HCC-targeting peptide (SFSIIHTPILPLGGC)
SSL: sterically stable liposomes
SSTRs: somatostatin receptors
SSTR2: somatostatin receptor subtype 2
SSTR5: somatostatin receptor subtype 5
SUVs: small unilamellar vesicles
TAA: thioacetamide
TAT: transactivator of transcription peptide
TF-TP@LIP: transferrin–triptolide liposomes
Tf-LP-ERN: transferrin–erianin liposomes
TfR: transferrin receptor
TfRs: transferrin receptors
TGFβ: transforming growth factor β
ThermoDox: thermosensitive liposomal doxorubicin formulation
TP: triptolide
ULVs: unilamellar vesicles
